# SOX4-STAT6-MTHFD2 axis drives hepatocellular carcinoma progression and treatment resistance

**DOI:** 10.1038/s41419-025-08394-2

**Published:** 2026-01-03

**Authors:** Chia-Lung Tsai, Ming-Chin Yu, Cheng-Lung Hsu, Hsiang-Yu Tang, Yun-Shien Lee, Lang-Ming Chi, Sey-En Lin, Mei-Ling Cheng, Heng-Yuan Hsu, Chi-Neu Tsai

**Affiliations:** 1 Genomic Medicine Core Laboratory, Linkou Chang-Gung Memorial Hospital, Taoyuan City, 33305 Taiwan; 2 Department of Surgery, New Taipei Municipal Tucheng Hospital (Built and operated by Chang-Gung Medical Foundation), New Taipei City, 23652 Taiwan; 3 School of Medicine, College of Medicine, Chang-Gung University, Taoyuan City, 33302 Taiwan; 4 Division of Hematology-Oncology, Department of Internal Medicine, Chang-Gung Memorial Hospital, Taoyuan City, 33305 Taiwan; 5 Metabolomics Core Laboratory, Healthy Aging Research Center, Chang Gung University, Taoyuan, 33302 Taiwan; 6 Department of Biotechnology, Ming Chuan University, Taoyuan, 33348 Taiwan; 7 Molecular Medicine Research Center, Chang-Gung University, Taoyuan City, 33302 Taiwan; 8 Department of Anatomical Pathology, Taipei Institute of Pathology, Taipei City, 10372 Taiwan; 9 Department of Biomedical Sciences, Chang-Gung University, Taoyuan City, 33302 Taiwan; 10 Graduate Institute of Clinical Medical Sciences, College of Medicine, Chang-Gung University, Taoyuan City, 33302 Taiwan

**Keywords:** Cancer metabolism, Liver cancer

## Abstract

Hepatocellular carcinoma (HCC) is a major global health burden. Despite recent advances in immunotherapy, tyrosine kinase inhibitors (TKIs) treatment or combined therapies, therapeutic resistance and disease progression remain significant challenges. SOX4, a transcription factor frequently overexpressed in HCC and other cancers, has been linked to drug resistance and poor prognosis; however, the underlying molecular mechanisms remain unexplored. In this study, we identify STAT6 as a novel transcriptional target and interacting partner of SOX4 in HCC cells. Genetic ablation or knockdown of SOX4 induced hypermethylation of the STAT6 promoter, suppressing its expression, while treatment with the DNA methyltransferase inhibitor 5-Aza-2’-deoxycytidine restored STAT6 levels, indicating an epigenetic mechanism of regulation. In addition, SOX4 is physically associated with STAT6, as confirmed by co-immunoprecipitation and immunofluorescence. SOX4 depletion impaired interleukin-4 (IL-4)-induced phosphorylation of STAT6 at tyrosine residue 641 (Y641), implicating SOX4 in IL-4-mediated STAT6 activation. Chromatin immunoprecipitation (ChIP) assays demonstrated that SOX4 and STAT6 co-occupy the promoter of *MTHFD2*, a key enzyme in folate metabolism, regulating NADH/NADPH production and nucleotide biosynthesis. Knockdown of SOX4 or STAT6, or mutation of their binding sites within the *MTHFD2* promoter, reduced *MTHFD2* expression, NADPH levels, and nucleotide synthesis. Transcriptomic analyses from TCGA-LIHC and our independent cohort revealed a strong positive correlation between SOX4, STAT6, and MTHFD2, with MTHFD2 overexpression linked to poor overall survival. Clinically, elevated SOX4/STAT6/MTHFD2 axis activity was associated with resistance to immunotherapy or TKIs, either in our enrolled cohort or transcriptome data obtained from GSE109211. Metabolomic profiling further revealed increased NADPH and nucleotide biosynthesis in tumors with high SOX4/STAT6/MTHFD2 expression. Targeting STAT6 or MTHFD2 suppressed tumor growth in TKIs-resistant patient-derived xenograft models. Collectively, our findings identify the SOX4–STAT6–MTHFD2 axis as a critical driver of HCC progression and therapeutic resistance, offering a promising target for intervention in refractory HCC.

## Introduction

Liver cancer is the sixth diagnosed malignancy and ranks as the third leading cause of cancer-related deaths globally [1]. Hepatocellular carcinoma (HCC) comprises approximately 90% of primary liver cancers and remains a significant global public health concern. Curative options for early-stage HCC include surgery, local ablation, or transplantation, but recurrence leads to poor 5-year survival rates [2, 3**]**. Advanced HCC is managed with embolization and targeted therapies like immune checkpoint inhibitors (ICIs), tyrosine kinase inhibitors (TKIs), or anti-angiogenic agents [2, 4, 5]. Despite therapeutic advancements, disease progression remains a concern, highlighting the need to identify critical targets to improve treatment efficacy.

SOX4, a member of the SOX family, contains the SRY-related high-mobility-group box, which binds to [(A/T)(T/A)CAA(A/T)G] DNA consensus sequences in promoters to regulate genes involved in neural development, sex determination, differentiation, epithelial–mesenchymal transition (EMT), and angiogenesis in cancers [6]. Its overexpression correlates with poor prognosis in multiple cancers, including HCC [7–16]. Recent studies suggest SOX4 as a potential biomarker for poor immunotherapy outcomes [17, 18]. Despite its role in tumor progression, SOX4 remains undruggable. Functionally, SOX4 acts as a hub to interact with proteins like EZH2, P53, Smad3, β-catenin, and TCF/LEF in response to various signaling [6]. Notably, the SOX4–EZH2 axis has been shown to drive metastasis via epigenetic reprogramming, including modulation of microRNAs and transcriptional/metabolic changes [8, 10]. In addition, SOX4 has also been reported to be associated with SMARCA4- the catalytic ATPase subunit of the SWI/SNF chromatin remodeling complex, to regulate transcriptional targets such as *TGFBR2* [19]. Collectively, these findings highlight a critical role for SOX4 in epigenetic regulation. Continued investigation into its regulation and interactome may uncover its role in carcinogenesis and therapeutic potential.

STAT6 is essential for M2 macrophage polarization, driven by IL-4/IL-13, which fosters tumor progression by creating an immunosuppressive microenvironment [20]. In the canonical IL-4/IL-13 pathway, these cytokines bind to interleukin-4 receptor alpha, activating Janus kinase 1, which phosphorylates STAT6 at tyrosine residue 641 (p-STAT6 Y641), prompting its dimerization and nuclear translocation to regulate gene expression [21]. Beyond phosphorylation, the STAT6 promoter is modulated by epigenetic modifications [22**–**24]. Recent therapeutic strategies target STAT6 in immune disorders and cancers [21, 25–27]. Besides, STAT6 overexpressed in various malignancies, including breast, pancreatic, prostate, and colorectal cancers, promotes macrophage activation and tumor metastasis [28**–**33]. Knockdown of STAT6 reduced HepG2 and Hep3B cell proliferation by suppressing nuclear factor kappa-B ligand (RANKL) expression [34]. However, its role in HCC carcinogenesis remains unclear due to limited studies.

Methylenetetrahydrofolate dehydrogenase 2 (MTHFD2) is a mitochondrial enzyme in one-carbon folate pathway, catalyzing the NAD + /NADP + - dependent conversion of 5,10-methylenetetrahydrofolate (CH_2_-THF) to 10-formyltetrahydrofolate (^10^CHO-THF) [35]. This reaction supports redox balance via the glutathione system and provides formate for purine biosynthesis [35]. CH2-THF also fuels thymidylate synthase (TYMS) in converting deoxyuridine monophosphate (dUMP) to deoxythymidine monophosphate (dTMP), essential for pyrimidine synthesis [35]. Besides, the interconnection between the folate pathway and the methionine cycle—where S-adenosylmethionine (SAM), a critical intermediate, serves as a universal methyl donor for DNA, RNA, and protein methylation [35]. Additionally, MTHFD2 promotes hexosamine biosynthesis pathway (HBP) to activate UDP-N-acetylglucosamine (UDP-GlcNAc) production, stabilizing c-Myc protein and enhancing PD-L1 expression, facilitating immune evasion in pancreatic cancer [36]. Besides, given its role in nucleotide metabolism, MTHFD2 drives cancer cell growth, stemness, drug resistance, and immune evasion, making it a promising therapeutic target for various kinds of cancers [37**–**39].

In our previous study, two SOX4 knockout cell lines (Hep3B SOX4^−/−^ #1 and #2) were previously generated using the clustered regularly interspaced short palindromic repeats and Cas9 (CRISPR/Cas9) system to investigate SOX4-regulated cellular pathways [11]. In this study, STAT6 was identified as a downstream regulatory protein of SOX4 in HCC cells through liquid chromatography–mass spectrometry (LC-MS) analysis. Therefore, we aimed to evaluate how SOX4 regulated STAT6, as well as their co-regulated genes and pathways, including the folate metabolism pathway and its key enzyme, MTHFD2, in HCC cells. The impact of the SOX4/STAT6/MTHFD2 axis on cellular metabolites, and prognosis of patients with HCC were also discussed in this study.

## Results

### SOX4 epigenetically regulated STAT6 expression in HCC cells

**Fig. 1:**
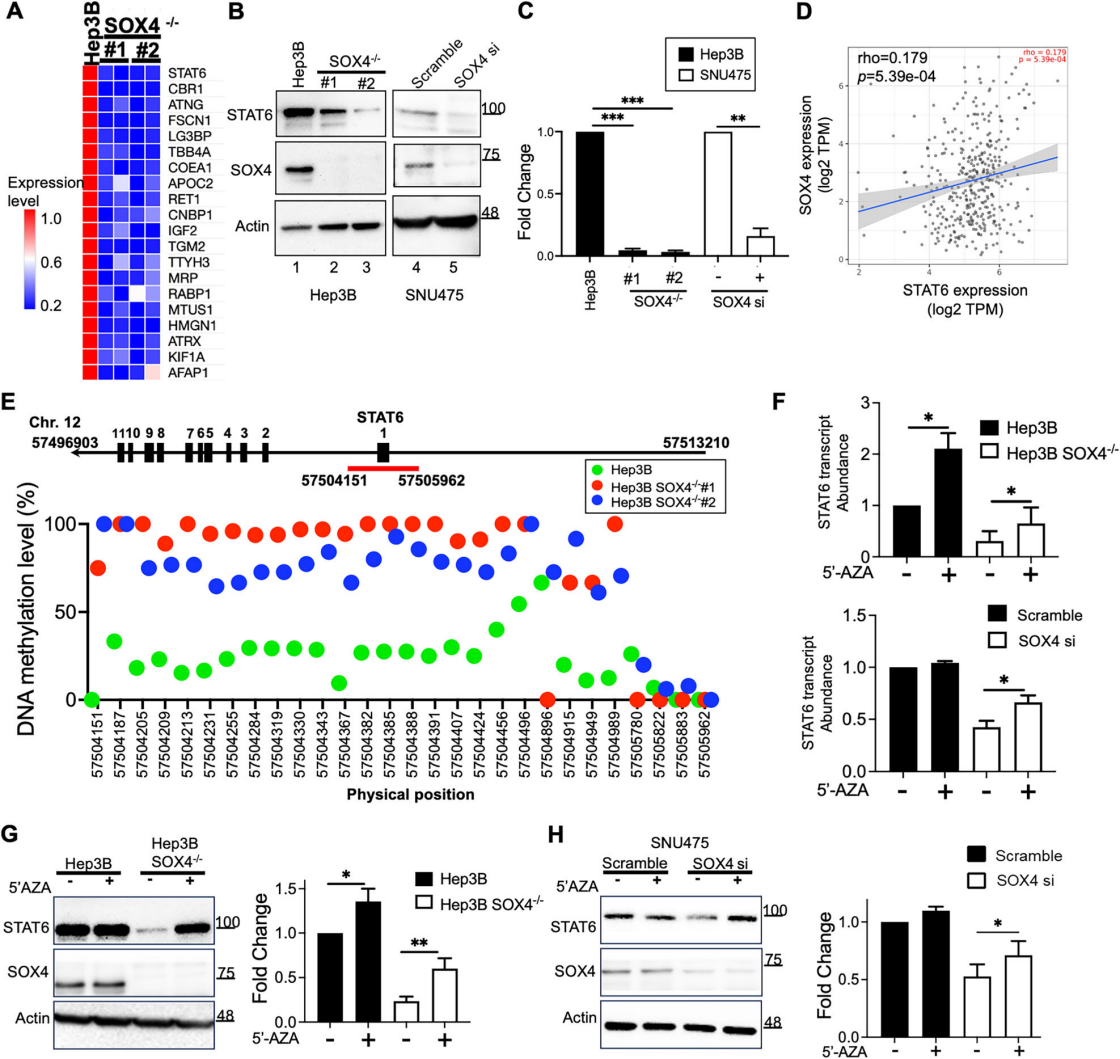
SOX4 epigenetically regulated STAT6 Expression in HCC Cells. **A** Differential protein expression in Hep3B and two SOX4 knockout (SOX4^−/−^ #1 and #2) cell lines was analyzed via tandem mass spectrometry [55]. The red indicated higher protein expression and blue indicated lower expression. The relative expression levels are represented by the scale bar. Protein expression in Hep3B cells was set as the baseline (1-fold). The corresponding protein expression in SOX4^−/−^ cells was normalized to the expression level in Hep3B cells and presented as a relative fold change. **B** Western blot analysis of SOX4 and STAT6 protein levels in Hep3B and Hep3B SOX4^−/−^ cells, as well as in SNU475 cells transfected with either scramble or SOX4 siRNA (si). Actin was used as an internal control. **C** STAT6 transcript levels were assessed using qRT-PCR in Hep3B and Hep3B SOX4^−/−^ cells, as well as in SNU-475 cells transfected with either scramble or SOX4 siRNA. 18S served as an internal control for normalization. STAT6 expression in Hep3B cells was set as the baseline (One-fold), and relative fold changes were determined in SOX4^−/−^ or SOX4 knockdown conditions. **D** Correlation analysis between SOX4 and STAT6 expression (*r* = 0.179, *p* = 5.39e-04) was performed using the TCGA-LIHC dataset on the TIMER2.0 website (http://timer.cistrome.org) with Pearson correlation. **E** The methylation status around the STAT6 exon 1 region (Chr12: 57504151-57505962) was analyzed via genomic DNA methylation sequencing in Hep3B and Hep3B SOX4^−/−^ cells. The methylation levels (%) of individual CpG sites were represented using green (Hep3B) or blue/red (Hep3B SOX4^−/−^) color spots. **F** qRT-PCR analysis of STAT6 transcript levels in Hep3B and Hep3B SOX4^−/−^ cells (upper panel) or SNU475 cells transfected with scramble or SOX4 si (lower panel) following treatment with or without 5-Aza-2’-deoxycytidine (5’-AZA). 18S was used as an internal control. The expression levels of STAT6 in Hep3B and SOX4^−/−^ cells, with or without treatment, were calculated using the ΔCt method. Specifically, the Ct value of STAT6 was normalized to that of the housekeeping gene 18S (ΔCt = Ct_STAT6 - Ct_18S). The transcripts of STAT6 in untreated Hep3B cells were set as the reference (one-fold). STAT6 expression in other conditions was then normalized to this reference and presented as relative STAT6 transcript abundance. **G**, **H** Western blot analysis of SOX4 and STAT6 protein levels in Hep3B and Hep3B SOX4^−/−^ cells (**G**) or in SNU475 cells (**H**) transfected with scramble or SOX4 si, with or without 5’-AZA treatment. Actin was used as an internal control. All Western blot and qRT-PCR analyses were performed in triplicate, and the results are presented as the mean ± standard deviation (SD) from three independent experiments (*P < 0.05, **P < 0.01).

To explore SOX4-regulated cellular pathways in HCC, its endogenous expression was assessed by western blotting across various liver cancer cell lines. SOX4 was highly expressed in Hep3B cells, moderately in Huh7 and SNU475, weakly in PLC5 and SNU398, and undetectable in HepG2 and two immortalized hepatocyte lines (Supplementary. Fig. 1). Based on this, two SOX4-knockout Hep3B cell lines (Hep3B SOX4⁻/⁻ #1 and #2), generated via CRISPR/Cas9 previously [11], were used to analyze SOX4-dependent protein changes using LC-MS. Among the differentially expressed proteins, STAT6 showed the most significant downregulation in SOX4 knockout cells (Fig. 1A). Results of western blotting and quantitative reverse transcription- real-time PCR (qRT-PCR) confirmed that repression of SOX4 reduced STAT6 at both protein (Fig. 1B, lane 2, 3 and lane 5) and mRNA levels in HCC cells (Fig. 1C), suggesting transcriptional regulation. Data from The Cancer Genome Atlas-Liver Hepatocellular Carcinoma Collection (TCGA-LIHC) supported a positive correlation between SOX4 and STAT6 transcripts (Fig. 1D). Although no SOX4 consensus binding sites were found in the STAT6 promoter, multiple CpG sites were located from the 5’-UTR to the first exon of STAT6 gene (Fig. 1E), suggesting potential epigenetic regulation. Given previous evidences implicating SOX4 in epigenetic regulation and reports of STAT6 promoter methylation [8, 10, 19, 22, 23], we next analyzed DNA methylation profiles in wild-type and SOX4-knockout Hep3B cells using bisulfite DNA sequencing. Bisulfite DNA sequencing showed increased methylation in the STAT6 5′-UTR to exon 1 in SOX4^−/−^ cells compared to wild-type (Fig. 1E). Treatment with the DNA methylation inhibitor 5-Aza-2′-deoxycytidine (5’-AZA) restored STAT6 mRNA **(**Fig. 1F**)** and protein levels (Fig. 1G, H) in SOX4-knockout/or knockdown cells, suggesting that SOX4 maintains STAT6 expression by preventing promoter methylation. These results indicate an epigenetic mechanism by which SOX4 regulates STAT6 in HCC cells.

### SOX4 complexed with STAT6 protein to modulate IL-4-mediated phosphorylation of STAT6 in HCC cells

**Fig. 2:**
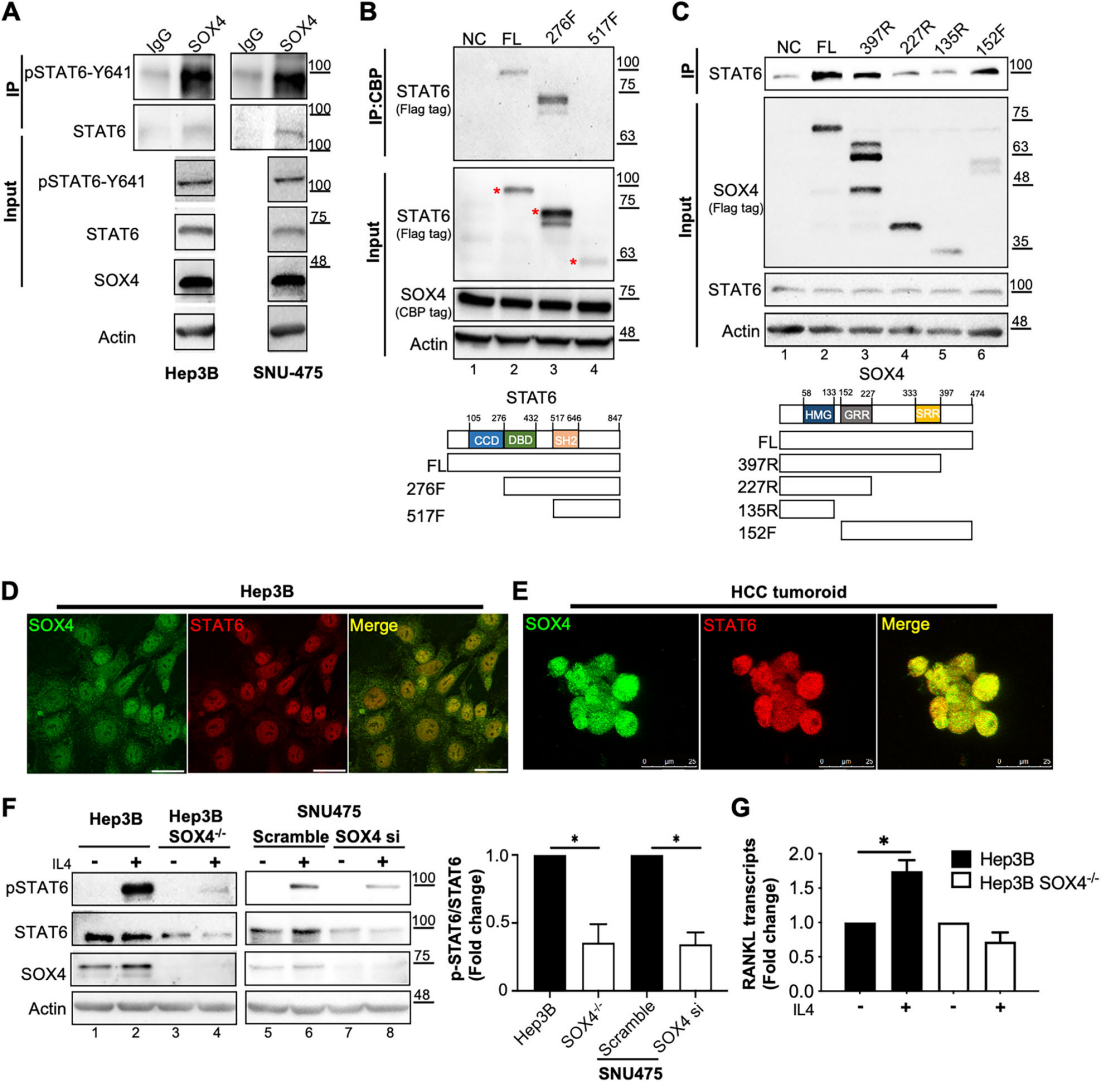
SOX4 Complexes with STAT6 in HCC Cells to Regulate IL-4-Mediated STAT6 Phosphorylation. **A** Immunoprecipitation (IP) assays were performed using 1 mg of Hep3B or SNU-475 cell lysates incubated with SOX4-specific or IgG control antibodies. Immunoprecipitated proteins were analyzed by western blotting for STAT6 and phosphorylated STAT6 (p-STAT6-Y641), with input lysates (1/20 volume) and actin serving as controls. **B** HEK293 cells were co-transfected with full length CBP-tagged SOX4 and STAT6 constructs, including various STAT6 deletion mutants. Co-immunoprecipitation was performed using CBP beads, followed by detection of the Flag-tagged STAT6 constructs with M2 (Flag) antibodies. Input protein (1/20 volume) expression was validated via western blotting. **C** Flag-tagged SOX4 deletion constructs were co-transfected with full-length STAT6 expression plasmids in HEK293 cells. SOX4 pull-downs were probed for STAT6, and western blotting confirmed input protein expression. **D**, **E** Immunofluorescence staining of fixed Hep3B cells (**D**) and HCC tumoroids (**E**) demonstrated colocalization of SOX4 and STAT6 in the nucleus. Secondary antibodies were conjugated with distinct colors, and localization was visualized by confocal microscopy. **F** Hep3B, Hep3B SOX4^−/−^, and SNU-475 cells transfected with scramble or SOX4 si were treated with IL-4. Western blot analysis was performed to assess p-STAT6-Y641, STAT6, and SOX4 expression, with actin as a loading control. After normalization with actin, IL-4-induced phosphorylation of p-STAT6-Y641 was quantified as p-STAT6/STAT6 (right panel), the ratio of p-STAT6-Y641/STAT6 in Hep3B or SNU475 cells treated with IL-4 was set as the baseline (1-fold). The corresponding protein expression in SOX4^−/−^ or SNU-475 transfected with SOX si cells was normalized to the expression level in Hep3B or SNU475 cells treated with IL-4 and presented as a relative fold change. **G** RANKL, an IL-4-STAT6 downstream gene, was examined by qRT-PCR by its specific primers in Hep3B and Hep3B SOX4^−/−^ cells treated with or without IL-4. 18S served as an internal control for gene expression normalization. The expression levels of RANKL in Hep3B and SOX4^−/−^ cells, with or without IL-4 treatment, were calculated using the ΔCt method. The Ct value of RANKL was normalized to that of the housekeeping gene 18S (ΔCt = Ct_RANKL - Ct_18S). The expression level of RANKL in untreated Hep3B cells was set as the reference (One-fold). RANKL expressions in other conditions were then normalized to this reference and presented as relative fold change. Western blot and qRT-PCR results were obtained from three independent experiments, with data presented as the mean ± SD (*P < 0.05, **P < 0.01, ***P < 0.001).

Since both SOX4 and STAT6 are transcription factors, their interaction was also examined via co-immunoprecipitation in HCC cells. SOX4 formed a complex with both total STAT6 and p-STAT6 Y641 (Fig. 2A), while IgG served as a negative control. Mapping of interaction domains using deletion constructs in HEK293 cells revealed that removal of the STAT6 DNA-binding domain (DBD) abolished its binding to SOX4 (Fig. 2B, lane 4), and deletion of the SOX4 serine-rich region (SRR) repressed its interaction with STAT6 (Fig. 2C, lanes 4–5). Confocal microscopy further confirmed nuclear colocalization of SOX4 and STAT6 in Hep3B cells (Fig. 2D) and HCC tumoroids (Fig. 2E).

To investigate the functional of the SOX4–STAT6 complex, Hep3B, Hep3B SOX4^−^^/−^, and SNU475 cells with scramble or SOX4 si transfection were treated with or without interleukin-4 (IL-4). IL-4 induced p-STAT6 Y641 in both HCC cell lines (Fig. 2F, lanes 2, 6), but this induction was reduced in SOX4-deficient cells (Fig. 2F, lanes 4, 8). The total STAT6 and p-STAT6/STAT6 ratio was reduced in Hep3B SOX4^−/−^ cells or SOX4 si-transfected SNU-475 cells, relative to their corresponding controls (Hep3B and scramble-transfected SNU-475, respectively. (Fig. 2F right panel). Furthermore, IL-4-induced expression of RANKL, a downstream STAT6 target, was diminished in SOX4-knockout Hep3B cells, as shown by qRT-PCR (Fig. 2G). These results indicate that SOX4 could modulate IL-4–mediated phosphorylation of STAT6 Y641 and its downstream expression of RANKL in Hep3B cells. Together, these results demonstrate that SOX4 physically interacts with STAT6 and colocalizes with STAT6 in the nucleus of HCC cells and tumoroids. Functionally, SOX4 is required for efficient IL-4–mediated phosphorylation of STAT6 at Y641 and for the subsequent induction of STAT6 target genes such as RANKL in HCC.

### SOX4 and STAT6 co-Regulate folate pathway in HCC Cells

**Fig. 3:**
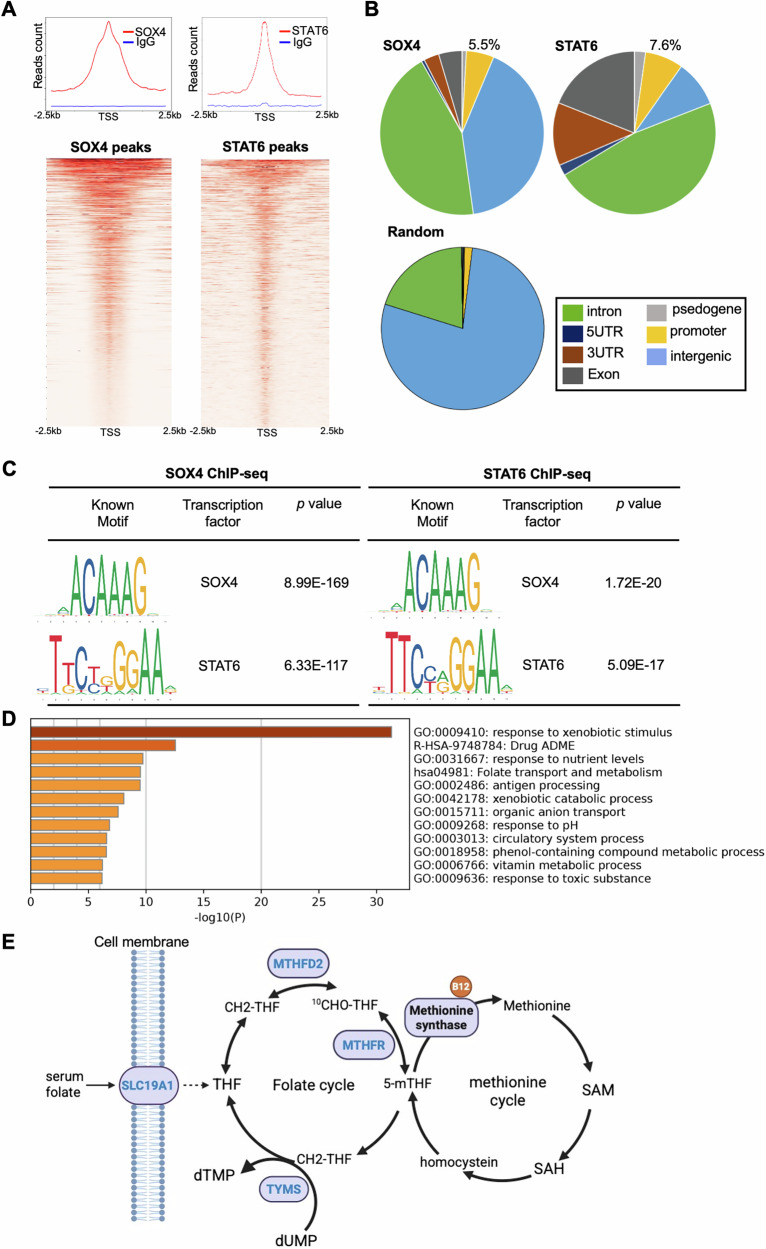
SOX4 and STAT6 co-regulated genes were identified by ChIP-sequencing in HCC cells. **A** Chromatin immunoprecipitation (ChIP) sequences were performed using SOX4, STAT6 antibodies or IgG on formaldehyde-fixed and fragmented chromatin from Hep3B cells using Cut and Run assay kit (Cell signaling). The ChIP sequencing analysis was performed by using a NovaSeq 6000, the peak call in the ChIP sequences were analyzed using Partek Flow (Illumina). The upper panel shows the average plot of SOX4, STAT6, or IgG signal in Hep3B cells. The lower panel displays occupancy maps of the top 800 genes, showing SOX4 and STAT6 binding regions within a 5 kb window around peak centers, with signal intensity as indicated. **B** The genome-wide distribution of SOX4 binding, STAT6 binding, or background genomic region (random). **C** Identification of SOX4 and STAT6 consensus DNA binding sequences in ChIP data using Partek Flow. **D** Gene ontology analysis of SOX4 and STAT6 co-bound genes in Hep3B cells using Metascape website. **E** The brief folate transport, metabolism, and the downstream methylation cycle. Genes co-regulated by SOX4 and STAT6, identified through ChIP-seq and RNA-seq, are highlighted in blue. Solute Carrier Family 19 (Folate Transporter), Member 1 (SLC19A1), Methylenetetrahydrofolate Reductase (MTHFR), Methylenetetrahydrofolate Dehydrogenase (NADP+ Dependent) 2 (MTHFD2), Thymidylate Synthetase (TYMS). This illustration was created with BioRender.com.

To identify genes co-regulated by SOX4 and STAT6, chromatin immunoprecipitation (ChIP) assays were performed in Hep3B cells using SOX4 and STAT6 antibodies, with IgG as a negative control. Both SOX4 and STAT6 antibodies showed enriched binding signals near peak centers compared to IgG (Fig. 3A), with 5.5% and 7.6% of peaks located in promoter regions; respectively (Fig. 3B). The identified consensus binding motifs matched known SOX4 and STAT6 sequences (Fig. 3C). Integration of ChIP-seq and RNA-seq data from Hep3B vs. SOX4^−/−^ cells and Hep3B scramble vs. STAT6 si knockdown cells identified 666 SOX4-regulated and 95 STAT6-regulated genes (Supplementary Fig. 2), with 86 overlapping targets (Supplementary Table 1). Furthermore, Gene Ontology analysis of the 86 shared targets showed enrichment in xenobiotic response and drug absorption, distribution, metabolism, and excretion (ADME) pathways, as well as folate-related metabolism (Fig. 3D). Notably, genes involved in folate transport and metabolism were enriched in SOX4 and STAT6 ChIP & RNA-seq. data (Fig. 3E), suggesting that SOX4 and STAT6 co-regulate pathways critical for drug metabolism and folate homeostasis in HCC cells. These results indicate that SOX4 and STAT6 co-occupy promoter regions and share a subset of transcriptional targets, with enrichment in folate-related metabolic pathways. This cooperative regulation highlights their role in modulating drug metabolism and folate homeostasis in HCC, we focused on their regulation of MTHFD2, a key enzyme in folate metabolism in this study.

### MTHFD2 was regulated by SOX4 and STAT6 in HCC cells

**Fig. 4:**
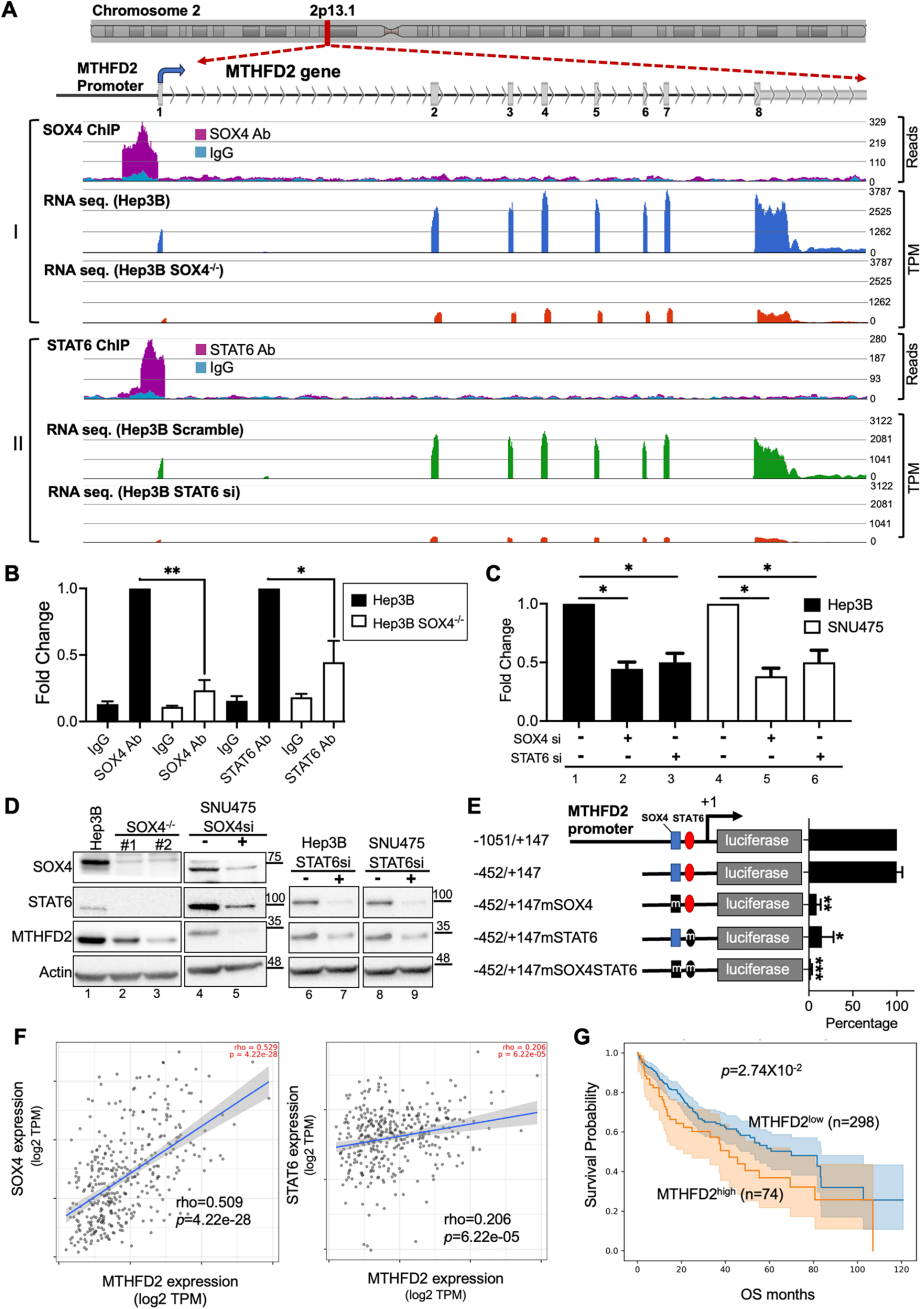
SOX4 and STAT6 Regulate MTHFD2 Expression in HCC Cells. **A** SOX4 and STAT6 ChIP-seq binding peaks at the MTHFD2 promoter. The upper schematic depicts genomic structure of the MTHFD2 gene (chromosome 2p13.1) including its exon 1 to 8 (gray rectangle), the transcription start site (blue arrowhead) and gene direction was as arrowhead indicated. Panel-I: SOX4-ChIP data showed binding peaks (purple) and IgG control (cyan), with the peak center is mapped within the MTHFD2 proximal promoter. The RNA-seq data comparing MTHFD2 gene expression between Hep3B (blue trace) and Hep3B SOX4^−/−^ cells (red trace) are shown as reads in Transcripts Per Million (TPM). Panel-II: STAT6-ChIP data (purple) and its IgG control (cyan) with the peak center enrichment within the MTHFD2 proximal promoter. Corresponding RNA-seq profiles compare MTHFD2 expression between Hep3B Scramble (green trace) and Hep3B STAT6-siRNA cells (red trace). **B** ChIP-qPCR validation of SOX4 and STAT6 binding at the MTHFD2 promoter in Hep3B versus Hep3B SOX4^−/−^ cells. Results were normalized to input genomic DNA and are presented as relative fold change. The ChIP-PCR results were based on three independent replicates. *P < 0.05, **P < 0.01. **C** MTHFD2 transcript levels in Hep3B and SNU475 cells transfected with Scramble, SOX4 si, or STAT6 si. Expression was normalized to 18S rRNA, with control (Scramble) set to 1X. The qRT-PCR data represent the mean ± SD from three independent experiments, *P < 0.05. **D** Western blot analysis of MTHFD2 protein levels: Hep3B and SOX4^−/−^ cells, as well as SNU475 cells transfected with SOX4 si (lane 1–5). Hep3B and SNU475 cells transfected with STAT6 si (lane 6-9). β-actin served as a loading control. **E** Schematic representation of SOX4 (blue squares) and STAT6 (red ovals) binding sites in the MTHFD2 promoter. Mutations in consensus sequences are marked as “m.” Reporter constructs containing these regions were cloned into a luciferase system and transfected into Hep3B cells. Luciferase activity was normalized to Renilla activity, with the −1051/ + 147 MTHFD2 reporter set as 100%. Data represent the mean ± SD from five independent experiments. (*P < 0.05, **P < 0.01). **F** Correlation analysis between SOX4 and MTHFD2 *(r* = 0.41, *P* = 0) and STAT6 and MTHFD2 (*r* = 0.33, *P* = 3.2 × 10⁻¹²*)* using TCGA-LIHC data on TMIER2.0, based on *P*earson correlation. **G** Kaplan–Meier survival analysis of HCC patients with high vs. low MTHFD2 transcript levels using TCGA-LIHC data.

Overexpression of MTHFD2 was associated with poor prognosis and drug resistance in various cancers [36**–**41]. ChIP-seq results confirmed SOX4 and STAT6 binding to the MTHFD2 proximal promoter (Fig. 4A), and RNA-seq data showed reduced MTHFD2 expression in SOX4^−/−^ (red trace) as compared to Hep3B cells (blue trace) (Fig. 4A-panel I), or STAT6 si-knockdown Hep3B (red trace) as compared with scramble-transfected cells (green trace) (Fig. 4A-panel II). ChIP-qPCR further validated SOX4 and STAT6 binding to the MTHFD2 proximal promoter, which was diminished in SOX4^−/−^ cells (Fig. 4B). MTHFD2 transcripts were decreased in Hep3B and SNU475 cells upon SOX4 or STAT6 knockdown as revealed by qRT-PCR (Fig. 4C, lane 2, 3 and lane 5, 6). Consistently, MTHFD2 protein levels were reduced in SOX4^−/−^ (lane 2, 3), SOX4 si- (lane 5) or STAT6 si-knockdown (lane 7, 9) HCC cells (Fig. 4D). These results suggest that SOX4 and STAT6 regulate MTHFD2 expressions at the transcriptional level.

Analysis of the MTHFD2 promoter revealed putative SOX4 (−181 to −174) and STAT6 (−152 to −140) binding sites within the proximal promoter (Fig. 4E). A series of promoter deletion constructs were cloned upstream of a TATA-less luciferase reporter to assess transcriptional activity. Mutation of either binding site reduced promoter activity, while dual mutations abolished it to basal levels (Fig. 4E). TCGA-LIHC data showed significant positive correlations between STAT6 and MTHFD2 (*p* = 6.22e−5, *r* = 0.206) and between SOX4 and MTHFD2 (*p* = 4.22e−28, *r* = 0.509) (Fig. 4F). Moreover, high MTHFD2 expression was associated with poorer overall survival in HCC patients (Fig. 4G). Collectively, these findings demonstrate that MTHFD2 is a transcriptional target of the SOX4–STAT6 complex, with both factors required for its promoter activity. Elevated MTHFD2 expression correlates with poor prognosis in HCC using TCGA-LIHC data.

### Role of SOX4/STAT6/MTHFD2 regulatory axis in NADH/NADPH production, purine synthesis in HCC cells

**Fig. 5:**
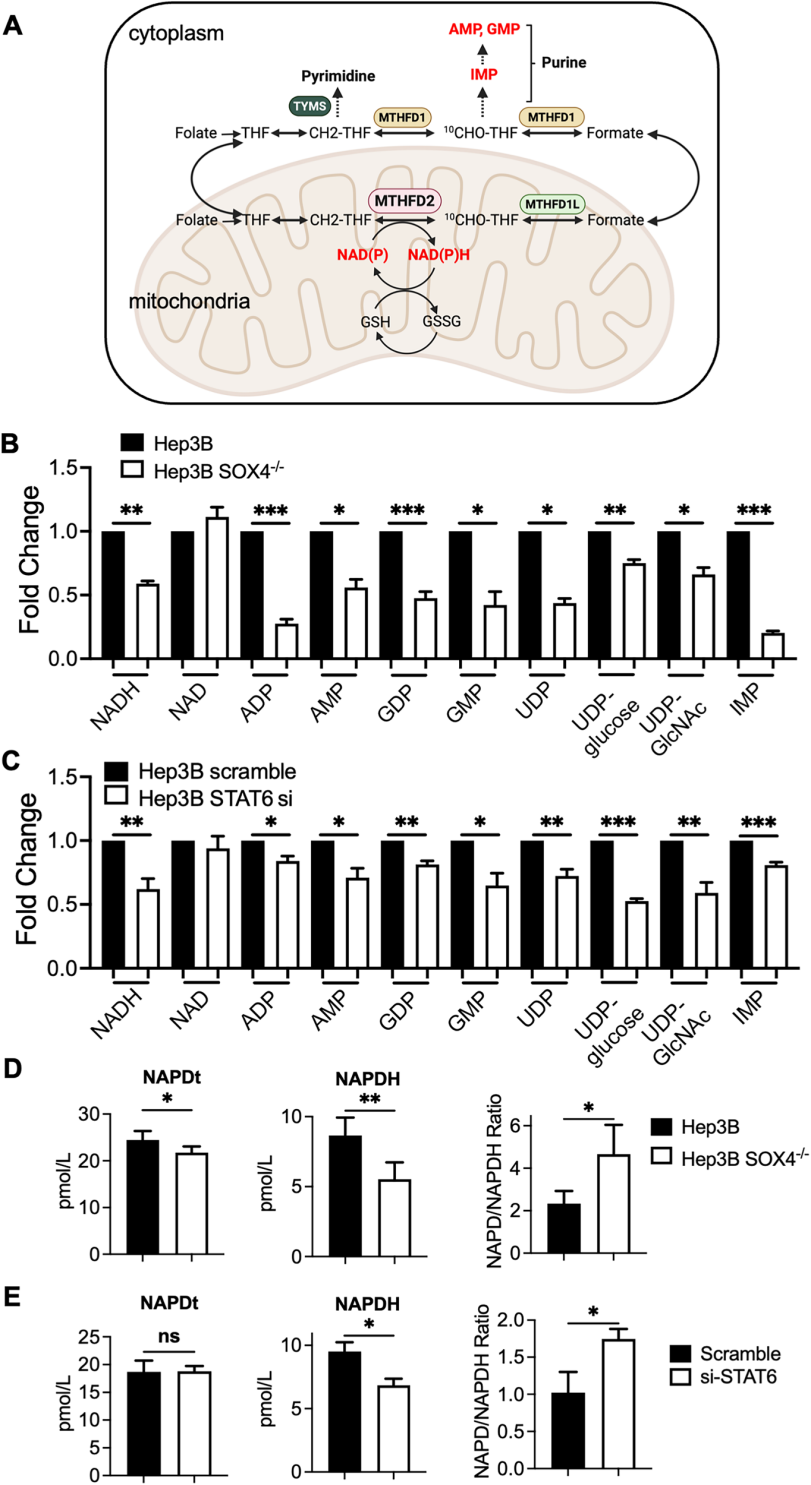
The production of NADPH, purine, hexosamine biosynthesis pathway (HBP) metabolites in SOX4 knockout cells or cells transfected with Scramble or si-STAT6. **A** Cytoplasmic and Mitochondrial One-Carbon Folate Cycle: Folate is first reduced to tetrahydrofolate (THF), which can be converted to 5,10-methylene-THF (CH2-THF) through enzymatic reactions. In the mitochondria, the bifunctional enzyme MTHFD2 (highlighted in pink) catalyzes the conversion of CH2-THF to 10-formyltetrahydrofolate (^10^CHO-THF), a reaction that requires NAD + /NADP+ and exhibits dehydrogenase activity. This conversion is crucial for maintaining redox balance, especially via the GSH/GSSG system. The ^10^CHO-THF produced is then converted to formate by MTHFD1L and transported to the cytoplasm. In the cytoplasm, MTHFD1 regenerates ^10^CHO-THF from formate, which is essential for purine biosynthesis. Additionally, in the cytoplasm, CH2-THF serves as a substrate for thymidylate synthase (TYMS), which catalyzes the conversion of deoxyuridine monophosphate (dUMP) to deoxythymidine monophosphate (dTMP), critical for pyrimidine synthesis [40, 60]. This schematic was created with BioRender.com. **B**, **C** The metabolites abundances of the NADH, NAD, purine, uridine metabolites were analyzed by LC-MS in Hep3B and SOX4^−/−^ cells (**B**), Hep3B Scramble and Hep3B STAT si transfected cells (**C**). The metabolites abundances were normalized to control (Hep3B or Hep3B Scramble as one-fold). The corresponding metabolites abundances in SOX4^−/−^ or STAT6 si transfected cells was normalized to control and presented as a relative fold change. Data represent the mean ± SD from three independent experiments. (*P < 0.05, **P < 0.01, ***P < 0.001). **D** The levels of NADP+ and NADPH were measured in Hep3B and *SOX4*^*−*/−^ cells using a commercial kit (Abcam). The NADP/NADPH ratio was calculated as (NADP + - NADPH)/NADPH. **E** Hep3B cells transfected with scramble or STAT6 si were analyzed for NADP+ and NADPH levels using the Abcam kit. Data represent the mean ± SD from three independent experiments. (ns: non-significant, *P < 0.05, **P < 0.01).

MTHFD2 is critical for NADH/NADPH production, nucleotide synthesis [35] and HBP pathway [36] (Fig. 5A). Since our results showed that SOX4 and STAT6 regulate MTHFD2, the related metabolites including NADH/NADPH, purine nucleotides, and HBP metabolites such as UDP, UDP-glucose, and UDP-GlcNAc, were analyzed using LC-MS in Hep3B versus Hep3B SOX4⁻/⁻ cells, as well as in scramble- versus STAT6 si-transfected Hep3B cells. In SOX4⁻/⁻ or STAT6 knockdown cells, levels of NADH, IMP, AMP, ADP, GMP, GDP, UDP, UDP-glucose, and UDP-GlcNAc were significantly reduced compared to Hep3B cells or scramble transfected cells, while NAD levels remained unchanged (Figs. 5B, C). Due to LC-MS sensitivity limits for NADPH, ELISA assays were used, revealing significantly decreased NADPH levels in SOX4^−/−^ (Fig. 5D) and STAT6-knockdown cells (Fig. 5E), with a corresponding increase in NADP/NADPH ratio. These results suggest that SOX4 and STAT6, through regulation of MTHFD2, promote NADH/NADPH production, de novo purine synthesis, and HBP metabolite generation in HCC cells.

### Expression of SOX4/STAT6/MTHFD2 in HCC specimen and their association with metabolite profiles and drug resistance

**Fig. 6:**
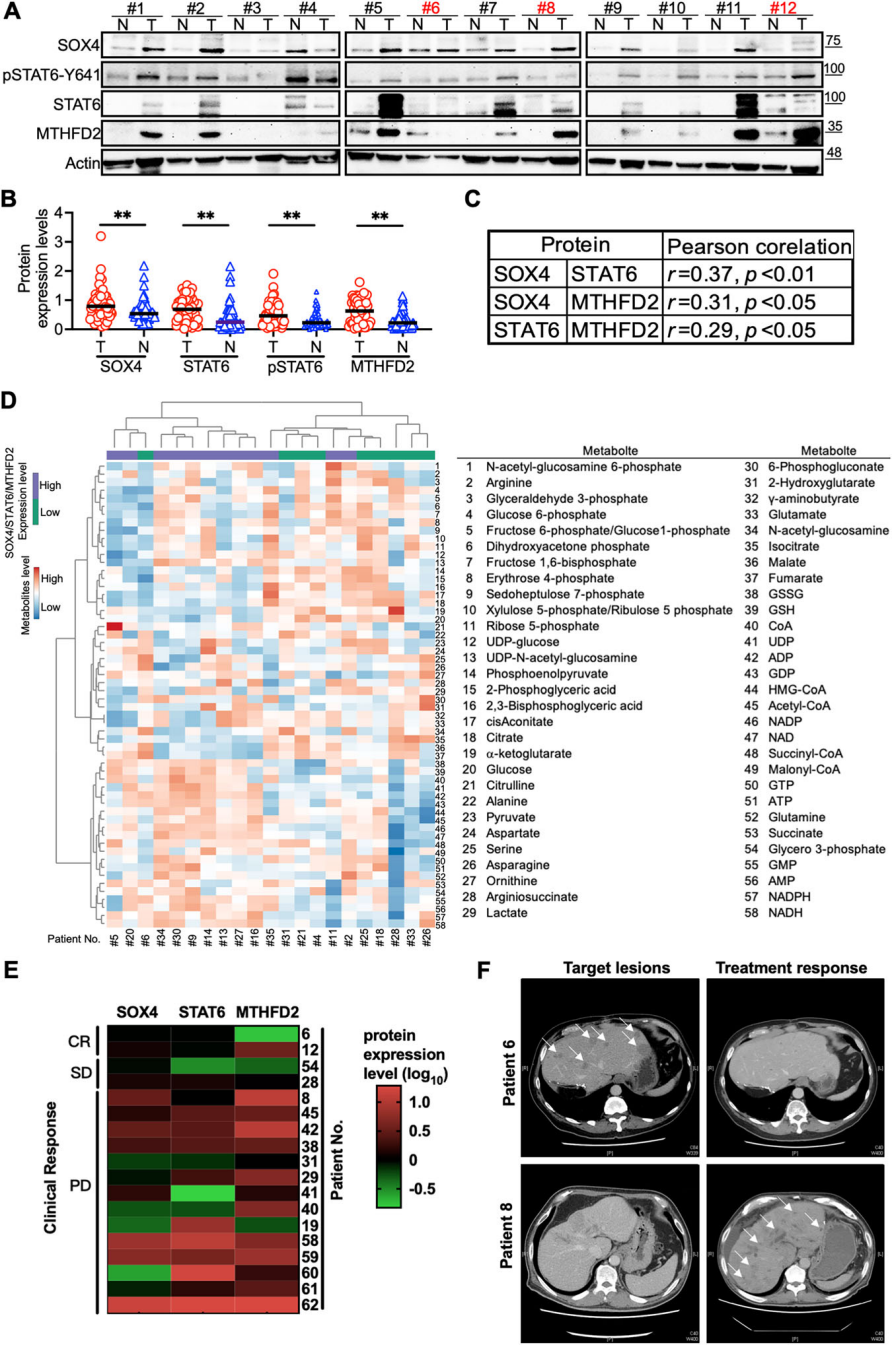
Expression of SOX4, STAT6, p-STAT6, and MTHFD2 in HCC Specimens and Their Impact on Metabolomics and Drug Resistance. **A** Western blot analysis of SOX4, p-STAT6 (Y641), STAT6, and MTHFD2 in paired normal (N) and tumor (T) tissues from 62 HCC patients. Representative data from 12 patients are shown; results for 50 additional samples are in Supplementart Fig. 3. β-actin was used as a loading control. Patients receiving TKIs, immunotherapy, or combination treatment are highlighted in red. **B** Quantification of protein band intensities using ImageJ software (NIH, Bethesda, MD, USA), normalized to β-actin. Statistical significance: *P < 0.05, **P < 0.01. **C** Correlation analysis of protein expression levels. **D** Metabolomic analysis of SOX4/STAT6/MTHFD2^high^ and SOX4/STAT6/MTHFD2^low^ tumor samples (*n* = 21) using LC-MS. Differential metabolite abundance was analyzed with MetaboAnalyst 6.0 website (https://www.metaboanalyst.ca/home.xhtml) [59]. The heatmap (left) shows log-ratio values, with red indicating upregulation and blue indicating downregulation. Identified metabolites are numbered 1-58 (right). The abundance of metabolites was showed in log_10_ value as scale bar. The sample number of clinical specimen was the same as the sample number present in Fig. 6A and Supplementary Fig. 3. **E** Clinical response and SOX4, STAT6, and MTHFD2 expression in 18 HCC specimens. Expression was normalized to β-actin, then further normalized as tumor/normal fold change. Colors indicate relative expressions (green to red). **F** Patient #6 (61 y/o male): Treated with atezolizumab and bevacizumab for 9 cycles, achieving near-complete response at 6 months, with treatment cessation at 8 months. Patient #8 (45 y/o male): Progressed despite sorafenib and transarterial chemoembolization.

To assess the clinical relevance of the SOX4/STAT6/MTHFD2 axis, we analyzed their protein expression in 62 HCC tumor and adjacent normal tissue pairs, with representative results from 12 pairs shown in Fig. 6A and the remaining 50 in Supplementary Fig. 3. The demographic data of the enrolled 62 cases was listed in Supplementary Table 2. Western blotting revealed significantly elevated levels of SOX4, STAT6, p-STAT6 (Y641), and MTHFD2 in tumor tissues compared to adjacent normal (Fig. 6B). Protein expression levels were positively correlated across the cohort (Fig. 6C). Immunohistochemistry further confirmed co-expression and spatial colocalization of SOX4, STAT6, and MTHFD2 in tumor lesions (Supplementary Fig. 4)

Based on protein expression, samples were stratified into SOX4/STAT6/MTHFD2^high^ (*n* = 12) and SOX4/STAT6/MTHFD2^low^ (*n* = 9) groups for metabolomic profiling via LC-MS. Partial Least Squares-Discriminant Analysis (PLS-DA) revealed clear separation between the two groups (Supplementary Fig. 5A) and volcano plot analysis identified significantly altered metabolites (Supplementary Fig. 5B). Heatmap analysis showed increased levels of MTHFD2-associated metabolites—including GSH, GSSG, AMP, GMP, NADH, NADPH, UDP, and UDP-glucose—in the SOX4/STAT6/MTHFD2^high^ group (Fig. 6D, metabolites 38–58).

We next examined the relationship between SOX4, STAT6, and MTHFD2 expression and treatment response in HCC patients. Among the 62 patients, 18 received (TKIs), immunotherapy, or combination therapies (Table 1). Protein levels of SOX4, STAT6, and MTHFD2 were quantified as relative folds (expression level in tumor/normal) and correlated with clinical response (Fig. 6E). Two patients achieved complete response (CR). Patient #6, who had multiple intrahepatic recurrences, showed complete tumor regression after nine cycles of atezolizumab and bevacizumab, maintaining CR for 18 months post-treatment (Table 1, Fig. 6F). Patient #12, treated with adjuvant sorafenib followed by surgical resection of peritoneal metastases, remained in CR for six months (Table 1). Both CR cases had low SOX4, STAT6, and MTHFD2 expressions compared to patients with progressive disease (PD) (Fig. 6E). Two patients (#54, #28) with stable disease (SD) also showed relatively low expression levels (Fig. 6E). In contrast, patient #8, who experienced disease progression following sorafenib and TACE, had high expression of all three proteins (Table 1, Fig. 6F). Analysis of transcriptomic data from GSE109211 further supported these findings, as elevated SOX4, STAT6, and MTHFD2 were observed in nearly half of sorafenib-nonresponsive lesions (22/46), but rarely in sorafenib-responsive tumors (Supplementary Fig. 6).

**Table 1:** The clinical treatment, drug responses, and treatment duration of patients with HCC.

Patient number	Target lesions	Treatment line	Combination	Outcome	Survival time (m)	DOT (m)	Clinical Response	Best clinical Response (BCR)
**#6**	Liver	AB	Nil	Alive, off treatment	25.3	8	CR	CR
**#12**	Peritoneum	S → R	Surgical resection	Alive, off treatment	24.2	12.4	CR	CR
**#54**	Liver	AB	RFA	Alive and treatment	8.7	8.7	SD	PR
**#28**	Liver, adrenal	S → P → L	Nil	Alive and treatment	20.3	20.3	SD	PR
**#8**	Liver	S → R	Nil	Death	15.8	15.8	PD	SD
#45*	Liver	AB	RFA, TACE	Death	5.1	5.1	PD	PD
**#42**	Liver, lymph nodes	DB	Nil	Death	24.5	24.5	PD	CR
**#38**	Liver	S → R	TACE	Death	6.8	6	PD	PD
**#31**	Liver	AB → N → S	Nil	Death	18	18	PD	PR
#29*	Liver, bone	P → N	TACE	Alive and treatment	10.7	10.7	PD	SD
**#41**	Lung	S	TACE	Death	0.4	0.4	PD	PD
**#40**	Peritoneum	S → R	Radiotherapy	Death	28.3	28.3	PD	SD
**#19**	Liver	AB → S → R → CT	Nil	Death	7.3	7.3	PD	PD
**#58**	Lung	S → R → S	Radiotherapy	Death	6.5	5.9	PD	SD
**#59**	Lung, bone	S → R	Surgical resection	Alive and treatment	16.9	16.9	PD	SD
**#60**	Liver	P → RM	Nil	Death	10.5	7.5	PD	PD
**#61**	Liver, lung	S → R → AB → CT	Nil	Death	26.1	24.1	PD	SD
**#62**	Liver, lymph nodes	AB → L → CT	Nil	Death	1.8	1.8	PD	PD

*DOT* duration of treatment, *m* months, *S* sorafenib, *R* regorafenib, *L* lenvatinib, *P* pembrolizumab, *N* nivolumab, *D* durvalumab, *A* atezolizumab, *B* bevacizumab, *RFA* radiofrequency ablation, *TACE* Transarterial chemoembolization, *CT* chemotherapy, *RM* Ramucirumab, *CR* complete response, *PR* partial response, *SD* stable disease, *PD* progressive disease. *female patient

patient number was the same as Fig. 6A and Supplementary Fig. 3 shown.

Taken together, these findings indicate that tumor tissues exhibited higher expression of SOX4, STAT6, and MTHFD2 associated with increased nucleotide synthesis, potentially contributing to drug resistance of patients with HCC.

### Targeting MTHFD2 or STAT6 inhibits cell growth and reduces tumor growth in a HCC PDx model

**Fig. 7:**
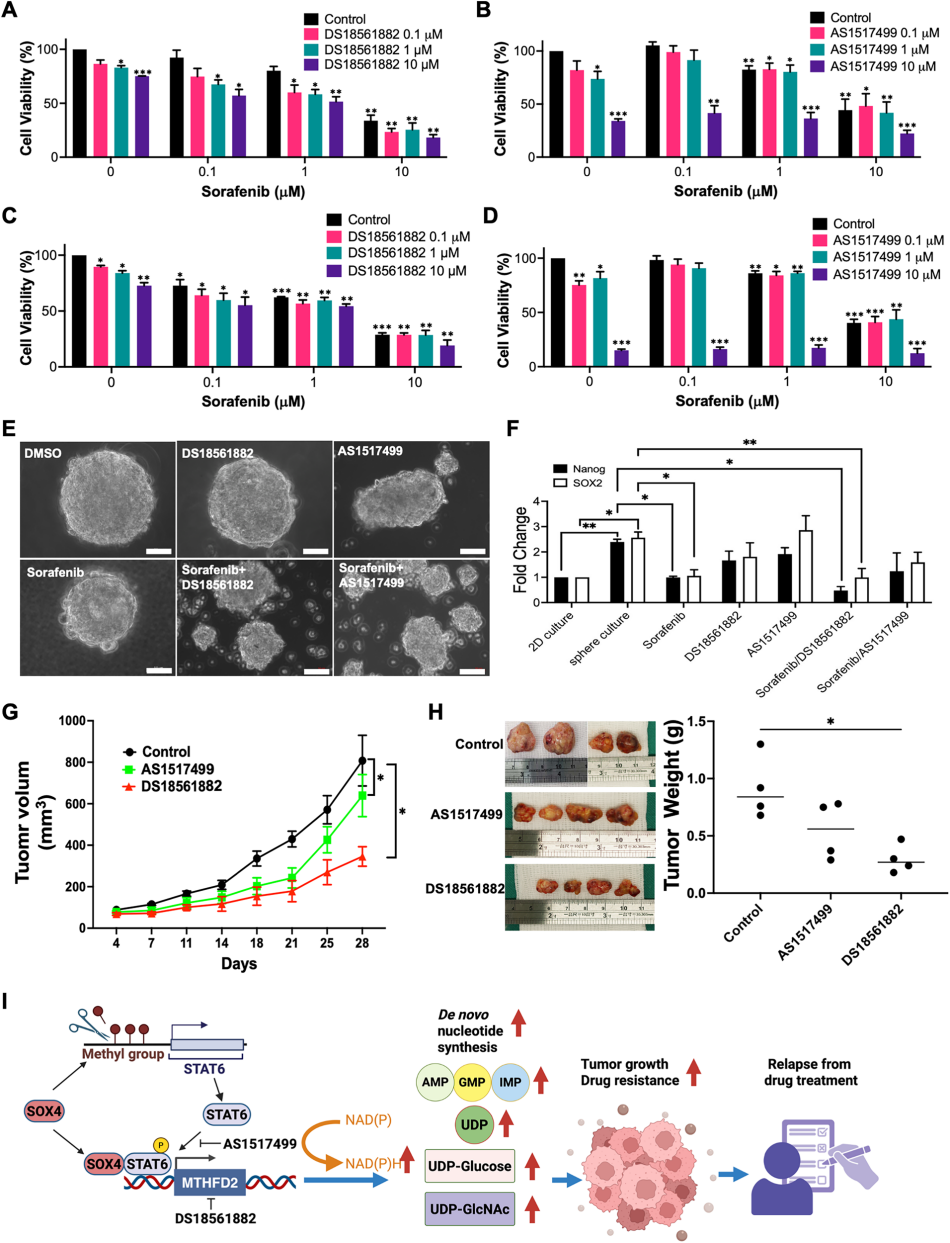
In Vitro and In Vivo Effects of STAT6 and MTHFD2 Inhibitors on HCC Growth. **A**, **B** Cell viability of Hep3B cells were treated with DS18561882 (MTHFD2 inhibitor) or AS1517499 (STAT6 inhibitor) with Sorafenib at various concentrations for 24 h. **C**, **D** Cell viability of SNU475 cells were treated with DS18561882 or AS1517499 in combination with sorafenib at various concentrations for 24 h. Cell viability was assessed using the CCK8 assay, with DMSO-treated cells defined as 100% viability (blue bar), and all other treatments were normalized accordingly and expressed as percentage viability. Data represents the mean ± SD from three independent experiments. *P < 0.05, **P < 0.01, ***P < 0.001. **E** Representative images of Hep3B sphere formation following treatment with DMSO, DS18561882, AS1517499, sorafenib, or their combinations. Scale bar, 50 μm. **F** The expressions of SOX2 and Nanog were accessed by qRT-PCR in sphere culture treated with DS18561882, AS1517499, or sorafenib or combination. Hep3B cells (5000 cells) were seeded in six-well plates coated with polypeptide polyelectrolyte multilayer films (Acrocyte Therapeutics, Taiwan) and cultured in sphere medium for 3 days, then cells were treated with DMSO, DS18561882, AS1517499, sorafenib, or their combinations. Spheres were harvested after 3 days of drug treatment for RNA extraction. Expression of stemness-associated genes was quantified by qPCR, normalized with expression level of 18S (internal control) firstly, then with Hep3B cells grown in 2D culture as baseline (normalized to 1×). The Y-axis shows relative fold-change in expression under sphere-forming conditions compared with 2D culture. Data represents the mean ± SD from three independent experiments. *P < 0.05, **P < 0.01. **G** PDX Tumor Growth: Tumors were excised from patients and implanted subcutaneously in five-week-old, anesthetized NPG mice. Inhibitors administered two weeks post-implantation that present as Day 0 in X axis, every 3–4 days through IP. Each treatment group included four mice. Tumor volume was measured as length × width × height (mm^3^). **H** The tumors were excised 28 days after drug treatment. The tumor weight (g) in control, AS1517499, DS18561882 treated group (right panel). Data represents the mean ± SD from four mice in each group. *P < 0.05. **I** Proposed model: SOX4 regulates STAT6 epigenetically and forms a complex with STAT6 to regulate MTHFD2, impacting de novo purine synthesis, NADPH production, and HBP metabolites, promoting tumor growth and drug resistance in HCC. This illustration was created with BioRender.com.

As SOX4 is not directly druggable, targeting its downstream effectors may offer therapeutic potential in SOX4-positive tumors. We assessed the effects of STAT6 (AS1517499) and MTHFD2 (DS18561882) inhibitors on cell viability in SOX4⁺ HCC cell lines. Given that previous studies reported enhanced sensitivity to TKIs or immunotherapy upon MTHFD2 inhibition [39], therefore combination of sorafenib with AS1517499 and DS18561882 were applied for examining cell viability in HCC cells as revealed by CCK8 assay. Treatment with AS1517499, DS18561882, or sorafenib alone significantly reduced cell proliferation in both Hep3B and SNU-475 cells (Fig. 7A–D). Notably, sorafenib combination with DS18561882 showed a synergistic effect in Hep3B cells (ZIP synergy score: 12.119) and an additive effect in SNU-475 cells (ZIP synergy score: 9.402) (Fig. 7A, C; Supplementary Fig. 7A, C). In contrast, sorafenib combined with AS1517499 did not produce synergy in either cell line (Fig. 7B, D; Supplementary Fig. 7B, D).

Consistent findings were observed in sphere formation assays using Hep3B cells. Spheres were treated with AS1517499, DS18561882, sorafenib, or their combinations (Fig. 7E), and stemness was assessed by qRT-PCR analysis of SOX2 and Nanog. As expected, SOX2 and Nanog expression was elevated when Hep3B cells transitioned from 2D to 3D culture (Fig. 7F). Importantly, DS18561882 combined with sorafenib suppressed both sphere growth and stemness marker expression more effectively than monotherapies, whereas AS1517499 did not exert a synergistic effect (Fig. 7F).

To evaluate in vivo efficacy, we employed an HBV⁺ HCC patient-derived xenograft (PDx) model from a 50-year-old male with advanced HCC. Histopathology confirmed a trabecular HCC architecture (Supplementary Fig. 8A). The patient’s tumor progressed rapidly despite sorafenib, and the PDx model (*n* = 3) demonstrated resistance to sorafenib, regorafenib, and ribavirin (Supplementary Fig. 8B). RNA-seq analysis showed elevated SOX4, MTHFD2 expression relative to GEPIA median levels; however, expression of STAT6 was slightly higher than median levels (Supplementary Fig. 8C). Treatment with AS1517499 or DS18561882 began on day 14 following PDx implantation in NPG mice (*n* = 4), both inhibitors significantly reduced tumor volume and weight over a 4-week treatment period (Figs. 7G, H).

These findings suggest that targeting STAT6 or MTHFD2 can inhibit cell proliferation in vitro and suppress tumor growth in a drug-resistant HCC PDx model, offering a promising strategy for SOX4⁺ HCC.

## Discussion

This study is the first to reveal a mechanistic link between SOX4, STAT6, and MTHFD2 in cancer. We identified a novel SOX4–STAT6–MTHFD2 regulatory axis that plays a critical role in HCC cells, influencing NADH/NADPH production, de novo nucleotide biosynthesis, cancer stemness, and patient prognosis (Fig. 7I**)**. SOX4, previously recognized as a key transcription factor in maintaining cellular stemness, was found to cooperate with STAT6 to transcriptionally regulate *MTHFD2*, an essential metabolic enzyme required for nucleotide synthesis and cell proliferation in this study. Notably, we demonstrated that SOX4 modulates STAT6 expression through both epigenetic mechanisms—by preventing promoter hypermethylation—and direct protein–protein interactions. Based on our data the SOX4/STAT6/MTHFD2 axis plays a role in modulating folate transport and methionine metabolism in HCC cells (Fig. 3), potential linking SOX4 loss to impaired one-carbon metabolism and possible secondary STAT6 promoter hypermethylation. However, the specific mechanism of demethylation is uncertain. SOX4 has been previously implicated in cancer cell proliferation and angiogenesis [6, 11, 12], its newly established connection to *MTHFD2* further underscores its pivotal role in promoting HCC progression and therapeutic resistance.

Among the components of this regulatory axis, STAT6 has been well-documented in macrophage activation [21], but its involvement in HCC tumorigenesis has been less explored [34]. Notably, therapeutic targeting of STAT6 expression by its specific anti-sense oligonucleotide in macrophages has been investigated in clinical trials for advanced HCC, showing promising anti-tumor effects [42]. Although the therapy mentioned above was targeted to macrophages instead of cancer cells, the treatment effect on cancer cells itself should be paid attention to since the expression of STAT6 protein was also overexpressed in tumor lesions of HCC (Fig. 6A). Our findings indicate that inhibition of p-STAT6 using AS1517499 reduced HCC cell growth in vitro (Fig. 7B-D). However, the effect on HCC PDx tumor growth was less pronounced compared to MTHFD2 inhibition (Figs. 7G, H). One possible explanation is that STAT6 transcript levels were not as much elevated in the HBV + HCC PDx model as compared with the median STAT6 transcript level from GEPIA (Supplementary Fig. 8C). Furthermore, while AS1517499 reduced Hep3B sphere size, it did not significantly downregulate the stemness markers SOX2 and Nanog, suggesting that inhibition of p-STAT6 may exert its effects through alternative, yet unidentified, pathways that require further investigation.

Several inhibitors have been developed to target MTHFD2 for cancer treatment based on its structure [43]. A study has shown that treatment with MTHFD2 inhibitors in acute myeloid leukemia slows the replication fork, increasing replication stress and inducing cellular apoptosis in vitro [37]. In HCC, MTHFD2 repression has been shown to inhibit cell growth and sensitize cells to TKIs [39]. Additionally, TP53 deficiency has been reported to trigger the transcriptional activation of MTHFD2, promoting cell proliferation [44]. TCGA-LIHC data indicate that TP53 mutations occur in approximately 32% of HCC specimens, though site-specific mutant effects [45, 46] on activation of MTHFD2 remain unclear. Our study’s limitation is that it did not assess the status of TP53 mutations in clinical samples. Besides, in folate pathway, 5-Fluorouracil (5-FU) is a chemotherapy drug that targets TYMS to reduce pyrimidine synthesis, which is used to treat various kinds of cancers [47], but HCC is considered as chemotherapy-resistant tumor, therefore 5-FU is not routinely administered [48]. However, dihydropyrimidine dehydrogenase (DPD) activity may predict 5-FU efficacy [49]. To assess whether simultaneous inhibition of TYMS and MTHFD2 could enhance therapeutic effects, we evaluated the combination of 5-FU with MTHFD2 or STAT6 inhibitors. In Hep3B cells, neither combination produced synergy (Supplementary Fig. 9A, B). By contrast, 5-FU combined MTHFD2 inhibition showed a synergistic effect in SNU-475 cells (Supplementary Fig. 9C), whereas no synergy was observed with 5-FU and STAT6 inhibition (supplementary Fig. 9D). Since DPD activity was not examined in these cells, the observed differences in responsiveness may reflect cell line–specific metabolic features and merit further investigation.

In conclusion, SOX4 epigenetically regulates STAT6 transcription and forms a complex with STAT6 to co-regulate MTHFD2 expression in HCC cells. This regulation enhances NADPH production, purine synthesis, and activation of the HBP pathway in tumors (Fig. 7I). Tumors with high SOX4/STAT6/MTHFD2 expression are more resistant to TKIs and immunotherapy, though our sample size was limited. However, analysis of transcriptomic data from GSE109211 supported our findings, as elevated SOX4, STAT6, and MTHFD2 were observed in nearly half of sorafenib-nonresponsive lesions, but rarely in sorafenib-responsive tumors. Besides, targeting STAT6 or MTHFD2 significantly reduces cell proliferation in vitro and attenuates tumor growth in sorafenib-resistant PDx models (Fig. 7I). Therefore, disrupting this regulatory axis may help restore therapeutic sensitivity in HCC patients, providing a potential strategy for improving treatment outcomes.

### Patients and methods

This study was conducted in accordance with the Declaration of Helsinki and all relevant institutional guidelines and regulations. The study protocol was approved by the Institutional Review Board of Chang Gung Memorial Foundation, Taiwan (approval numbers: 202100994B0, 202200985B0, and 202202182B0). A total of 62 patients (Supplementary Table 2) who underwent curative-intent surgical resection for hepatocellular carcinoma at Chang Gung Memorial Hospital (CGMH), Linkou or New Taipei Municipal Tucheng Hospital were retrospectively enrolled. Written informed consent was obtained from all participants prior to tissue collection and data analysis. Patients aged 20–85 years were included, and individuals with a history of other malignancies were excluded.

Paired tumor and adjacent non-tumorous liver tissues (*n* = 62) were obtained and processed by the CGMH Research Specimen Processing Laboratory. Clinical and pathological data were collected retrospectively and analyzed in an anonymized manner. All downstream protein expression analyses and molecular studies were performed using de-identified samples. The clinical images presented in Fig. 6F are non-identifiable and contain no personal or identifying information. Therefore, additional consent for publication of images was not required. However, the written informed consent for publication of these images was obtained from patient #6; for patient #8, who passed away prior to manuscript preparation, publication consent was provided by his legally authorized representative.

### Cell lines and three-dimensional (3D) culture

Cell lines used in this study are listed in Supplementary Table 3. All were maintained per provider instructions. Hep3B SOX4^−/−^ cells [11] were cultured in complete medium with 4 ng/ml puromycin (Thermo Scientific). For 3D culture, 5000 cells were seeded in six-well plates coated with polypeptide polyelectrolyte multilayer films (Acrocyte Therapeutics, Taiwan) [50] The complete culture medium was supplemented with 1× B27 (Thermo Fisher Scientific), 20 ng/mL EGF (Thermo Fisher Scientific), and 20 ng/mL basic FGF (Sigma-Aldrich) for sphere culture.

### HCC tumoroid culture

Liver cancer tumoroids were isolated and cultured using a previously described method [51]. About 1 cm³ of liver cancer tissue was minced, digested at 37 °C for 1–2 h, filtered through a 70 µm strainer, and centrifuged at 300–400 g for 5 min. After PBS washing, cells were cultured in a 24-well organoid culture plate (Acrocyte Therapeutics, Taiwan).

### Genome DNA methylation sequence

Genomic DNA (500 ng) was fragmented (150–200 bp) (Covaris S2, Covaris Inc., USA), processed with the TruSeq® Methyl Capture EPIC Kit (Illumina), and enriched for 3.3 M CpG sites. Bisulfite conversion and PCR amplification were performed with Kapa HiFi Uracil+ polymerase. Libraries were assessed by Qubit and Bioanalyzer and sequenced on a NovaSeq 6000. Data processing followed [52] and was deposited on Gene Expression Omnibus (GEO) with accession number GSE286327.

### RNA sequencing

RNA-seq was conducted at the CGMH Genomic Medicine Research Core [53]. Data from Hep3B vs. Hep3B SOX4⁻/⁻ (#1, #2) and Hep3B/Scramble vs. Hep3B/STAT6 siRNA were deposited in GEO (GSE277540).

### Immunofluorescence staining

The immunofluorescence staining was as previously described [54] and the antibodies used in this study were listed in Supplementary Table 4. The slides were examined under a Leica TCS SP2 confocal laser scanning microscope (Leica Microsystems GmbH, Wetzlar, Germany).

### Immunohistochemistry (IHC)

The procedure of IHC staining was as previously described [11] and the antibodies used for IHC are listed on listed in Supplementary Table 4.

### Western blotting and co-immunoprecipitation

The western blotting and co-immuno-precipitation procedures were as previously described [55] and the primary antibodies used in this study were listed in Supplementary Table 4.

### Quantitative reverse-transcription polymerase chain reaction (qRT-PCR)

RNA was extracted with TRIzol (Thermo Fisher Scientific, 15596026), reverse transcribed, and analyzed by qPCR as described [11] using primers in Supplementary Table 5.

### Chromatin immunoprecipitation (ChIP) sequence

CUT&RUN (Cell Signaling, 86652) was performed on 1 × 10⁵ Hep3B or Hep3B SOX4^−/−^ cells with anti-SOX4 (Abnova, PAB14092). SOX4–DNA complexes were digested by pAG-MNase, purified, followed by library construction with a DNA library prep kit (Cell Signaling, #56795 and #29580) and sequenced (150 bp paired-end reads; NovaSeq 6000). Data were processed in Partek Flow and were deposited in GEO (GSE277728).

### Luciferase assay

Cells were transfected with luciferase and Renilla (1/20 of luciferase) reporters (Lipofectamine 3000, Thermo Fisher, L3000015). Activity was measured at 24 h using Dual-Glo® (Promega, E2920) and normalized to Renilla.

### NADPH/NADP assay

Total NADP and NADPH levels in HCC cells were measured using the NADP/NADPH Assay Kit (Abcam, ab65349) per the manufacturer’s instructions. Values were determined from a standard curve and expressed as pmol/well. The NADP total (NADPt)/NADPH ratio was calculated as (NADPt-NADPH)/NADPH.

### Cell proliferation assay

Hep3B cells (1 × 10⁴/per 96 well) were treated with AS1517499 (HY-100614, MedChemExpress, USA), DS18561882 (HY-130251, MedChemExpress, USA), sorafenib (HY-10201, MedChemExpress, USA), or 5-FU (Nang-Kuang pharmaceutical Co.). DMSO served as control. Viability was measured with CCK-8 (Biotools, Taiwan). Synergy was assessed by the ZIP model (SynergyFinder 3.0) [56].

### HCC Patient-derived xenograft (PDx) models

HCC PDx models were established as described [57]. Tumor specimens from metastatic HCC patients were cut into 3–5 mm pieces and implanted subcutaneously into anesthetized 5-week-old male NPG mice (NOD.Cg-Prkdcscid Il2rgtm1Vst/Vst, BioLASCO Taiwan), which were maintained under specific pathogen-free conditions under an approved protocol (2015121808) at the CGMH Laboratory Animal Center, Taiwan. The study comprised three groups (Vehicle, AS1517499, and DS18561882), with four mice per group. Mice were randomly assigned to the Vehicle, AS1517499, or DS18561882 treatment groups using a simple randomization procedure. All animals were processed in parallel under the same technician, housing, and handling conditions to minimize bias. AS1517499 (20 mg/mL) and DS18561882 (40 mg/mL) were injected intraperitoneally (0.1 mL) twice weekly from day 14 for 4 weeks. The investigators were not blinded to group allocation during treatment or outcome assessment. The tumor size measurements using calipers were objective and automated, minimizing investigator bias.

### Protein identification by tandem mass spectrometry

Protein identification was performed at the Clinical Proteomics Core Laboratory of CGMH, Linkou. Peptide spectra analysis followed established procedures [55].

### Cellular metabolites analyzed via LC/MS

Soluble metabolites in HCC cells were analyzed at the Core Laboratory of the Healthy Aging Research Center, Chang-Gung University, following a previously described protocol [58]. Data analysis was conducted using MetaboAnalyst 6.0 [59].

### Statistical Analysis

Analyses were performed in GraphPad Prism 9; Student’s *t* test for quantitative data. Overall survival was analyzed by Kaplan–Meier in SPSS 21. *P* < 0.05 was considered significant. Transcripts of SOX4, STAT6, MTHFD2 was download from GSE109211 dataset. The transcript data of SOX4, STAT6 and MTHFD2 in tumor lesions was normalized as percentile gene expression for statistical analysis.

## Supplementary information

Supplementary Figures and Tables

Original data-1

original data-2

## Data Availability

All supporting data are provided within the main text and supplementary materials. The high-throughput datasets generated in this study—including DNA methylation, ChIP-seq, and RNA-seq profiles—have been deposited in the GEO under accession numbers GSE286327, GSE277540, and GSE277728, respectively.

## References

[1] Bray F, Laversanne M, Sung H, Ferlay J, Siegel RL, Soerjomataram I, et al. Global cancer statistics 2022: GLOBOCAN estimates of incidence and mortality worldwide for 36 cancers in 185 countries. CA Cancer J Clin. 2024;74:229–63. 38572751 10.3322/caac.21834

[2] Brown ZJ, Tsilimigras DI, Ruff SM, Mohseni A, Kamel IR, Cloyd JM, et al. Management of hepatocellular carcinoma: a review. JAMA Surg. 2023;158:410–20. 36790767 10.1001/jamasurg.2022.7989

[3] Hsu HY, Tang JH, Huang SF, Huang CW, Lin SE, Huang SW, et al. Recurrence pattern is an independent surgical prognostic factor for long-term oncological outcomes in patients with hepatocellular carcinoma. Biomedicines. 2024;12. 10.3390/biomedicines12030655 PMC10968336 38540268

[4] Falette Puisieux M, Pellat A, Assaf A, Ginestet C, Brezault C, Dhooge M, et al. Therapeutic management of advanced hepatocellular carcinoma: an updated review. Cancers (Basel). 2022;14. 10.3390/cancers14102357 PMC9139863 35625962

[5] Haber PK, Puigvehi M, Castet F, Lourdusamy V, Montal R, Tabrizian P, et al. Evidence-based management of hepatocellular carcinoma: systematic review and meta-analysis of randomized controlled trials (2002-2020). Gastroenterology. 2021;161:879–98. 34126063 10.1053/j.gastro.2021.06.008 PMC12276942

[6] Moreno CS. SOX4: The unappreciated oncogene. Semin Cancer Biol. 2020;67:57–64. 31445218 10.1016/j.semcancer.2019.08.027 PMC7043201

[7] Guan Y, Jiang SR, Liu JG, Shi JR, Liu ZB. USP20 regulates the stability of EMT transcription factor SOX4 and influences colorectal cancer metastasis. Pathol Res Pract. 2022;233:153879. 35405623 10.1016/j.prp.2022.153879

[8] Li L, Liu J, Xue H, Li C, Liu Q, Zhou Y, et al. A TGF-beta-MTA1-SOX4-EZH2 signaling axis drives epithelial-mesenchymal transition in tumor metastasis. Oncogene. 2020;39:2125–39. 31811272 10.1038/s41388-019-1132-8

[9] Song GD, Sun Y, Shen H, Li W. SOX4 overexpression is a novel biomarker of malignant status and poor prognosis in breast cancer patients. Tumour Biol. 2015;36:4167–73. 25592378 10.1007/s13277-015-3051-9

[10] Tiwari N, Tiwari VK, Waldmeier L, Balwierz PJ, Arnold P, Pachkov M, et al. Sox4 is a master regulator of epithelial-mesenchymal transition by controlling Ezh2 expression and epigenetic reprogramming. Cancer Cell. 2013;23:768–83. 23764001 10.1016/j.ccr.2013.04.020

[11] Tsai CN, Yu SC, Lee CW, Pang JS, Wu CH, Lin SE, et al. SOX4 activates CXCL12 in hepatocellular carcinoma cells to modulate endothelial cell migration and angiogenesis in vivo. Oncogene. 2020;39:4695–710. 32404985 10.1038/s41388-020-1319-z

[12] Vervoort SJ, de Jong OG, Roukens MG, Frederiks CL, Vermeulen JF, Lourenco AR, et al. Global transcriptional analysis identifies a novel role for SOX4 in tumor-induced angiogenesis. Elife. 2018;7. 10.7554/eLife.27706 PMC6277201 30507376

[13] Vervoort SJ, Lourenco AR, Tufegdzic Vidakovic A, Mocholi E, Sandoval JL, Rueda OM, et al. SOX4 can redirect TGF-beta-mediated SMAD3-transcriptional output in a context-dependent manner to promote tumorigenesis. Nucleic Acids Res. 2018;46:9578–90. 30137431 10.1093/nar/gky755 PMC6182182

[14] Wang D, Hao T, Pan Y, Qian X, Zhou D. Increased expression of SOX4 is a biomarker for. malignant status and poor prognosis in patients with non-small cell lung cancer. Mol Cell Biochem. 2015;402:75–82. 25567207 10.1007/s11010-014-2315-9

[15] Wang L, Li Y, Yang X, Yuan H, Li X, Qi M, et al. ERG-SOX4 interaction promotes. epithelial-mesenchymal transition in prostate cancer cells. Prostate. 2014;74:647–58. 24435928 10.1002/pros.22783

[16] Wang L, Zhang J, Yang X, Chang YW, Qi M, Zhou Z, et al. SOX4 is associated with poor prognosis in prostate cancer and promotes epithelial-mesenchymal transition in vitro. Prostate Cancer Prostatic Dis. 2013;16:301–7. 23917306 10.1038/pcan.2013.25

[17] Deng X, Wang Y, Guo H, Wang Q, Rao S, Wu H. Pan-cancer analysis and experimental. validation of sox4 as a potential diagnosis, prognosis, and immunotherapy biomarker. Cancers (Basel). 2023;15. 10.3390/cancers15215235 PMC10649301 37958409

[18] Wu J, Zhu MX, Li KS, Peng L, Zhang PF. Circular RNA drives resistance to anti-PD-1. immunotherapy by regulating the miR-30a-5p/SOX4 axis in non-small cell lung cancer. Cancer Drug Resist. 2022;5:261–70. 35800365 10.20517/cdr.2021.100 PMC9255236

[19] Mehta GA, Angus SP, Khella CA, Tong K, Khanna P, Dixon SAH, et al. SOX4 and. SMARCA4 cooperatively regulate PI3k signaling through transcriptional activation of TGFBR2. NPJ Breast Cancer. 2021;7:40. 33837205 10.1038/s41523-021-00248-2 PMC8035213

[20] Maier E, Duschl A, Horejs-Hoeck J. STAT6-dependent and -independent mechanisms in Th2 polarization. Eur J Immunol. 2012;42:2827–33. 23041833 10.1002/eji.201242433 PMC3557721

[21] Karpathiou G, Papoudou-Bai A, Ferrand E, Dumollard JM, Peoc’h M. STAT6: a review of a signaling pathway implicated in various diseases with a special emphasis in its usefulness in pathology. Pathol Res Pract. 2021;223:153477. 33991851 10.1016/j.prp.2021.153477

[22] Goenka S, Kaplan MH. Transcriptional regulation by STAT6. Immunol Res. 2011;50:87–96. 21442426 10.1007/s12026-011-8205-2 PMC3107597

[23] Kim EG, Shin HJ, Lee CG, Park HY, Kim YK, Park HW, et al. DNA methylation and not allelic variation regulates STAT6 expression in human T cells. Clin Exp Med. 2010;10:143–52. 19949830 10.1007/s10238-009-0083-8

[24] Park SJ, Kim H, Kim SH, Joe EH, Jou I. Epigenetic downregulation of STAT6 increases. HIF-1alpha expression via mTOR/S6K/S6, leading to enhanced hypoxic viability of glioma cells. Acta Neuropathol Commun. 2019;7:149. 31530290 10.1186/s40478-019-0798-z PMC6747735

[25] Binnemars-Postma K, Bansal R, Storm G, Prakash J. Targeting the Stat6 pathway in tumor-associated macrophages reduces tumor growth and metastatic niche formation in breast cancer. FASEB J. 2018;32:969–78. 29066614 10.1096/fj.201700629R

[26] He K, Barsoumian HB, Puebla-Osorio N, Hu Y, Sezen D, Wasley MD, et al. Inhibition of STAT6 with antisense oligonucleotides enhances the systemic antitumor effects of radiotherapy and anti-PD-1 in metastatic non-small cell lung cancer. Cancer Immunol Res. 2023;11:486–500. 36700864 10.1158/2326-6066.CIR-22-0547 PMC10099280

[27] Lee YJ, Kim K, Kim M, Ahn YH, Kang JL. Inhibition of STAT6 Activation by AS1517499. Inhibits expression and activity of PPARgamma in macrophages to resolve acute inflammation in mice. Biomolecules. 2022;12. 10.3390/biom12030447 PMC8946515 35327639

[28] Chen J, Gong C, Mao H, Li Z, Fang Z, Chen Q, et al. E2F1/SP3/STAT6 axis is required for. IL-4-induced epithelial-mesenchymal transition of colorectal cancer cells. Int J Oncol. 2018;53:567–78. 29901191 10.3892/ijo.2018.4429 PMC6017240

[29] Cui X, Zhang L, Luo J, Rajasekaran A, Hazra S, Cacalano N, et al. Unphosphorylated. STAT6 contributes to constitutive cyclooxygenase-2 expression in human non-small cell lung cancer. Oncogene. 2007;26:4253–60. 17237818 10.1038/sj.onc.1210222

[30] Fu C, Jiang L, Hao S, Liu Z, Ding S, Zhang W, et al. Activation of the IL-4/STAT6 Signaling Pathway Promotes Lung Cancer Progression by Increasing M2 Myeloid Cells. Front Immunol. 2019;10:2638. 31798581 10.3389/fimmu.2019.02638 PMC6863933

[31] Li BH, Yang XZ, Li PD, Yuan Q, Liu XH, Yuan J, et al. IL-4/Stat6 activities correlate with apoptosis and metastasis in colon cancer cells. Biochem Biophys Res Commun. 2008;369:554–60. 18294957 10.1016/j.bbrc.2008.02.052

[32] Shi JH, Liu LN, Song DD, Liu WW, Ling C, Wu FX, et al. TRAF3/STAT6 axis regulates macrophage polarization and tumor progression. Cell Death Differ. 2023;30:2005–16. 37474750 10.1038/s41418-023-01194-1 PMC10406838

[33] Wang N, Tao L, Zhong H, Zhao S, Yu Y, Yu B, et al. miR-135b inhibits tumour metastasis in prostate cancer by targeting STAT6. Oncol Lett. 2016;11:543–50. 26870245 10.3892/ol.2015.3970 PMC4727074

[34] Qing T, Yamin Z, Guijie W, Yan J, Zhongyang S. STAT6 silencing induces hepatocellular carcinoma-derived cell apoptosis and growth inhibition by decreasing the RANKL expression. Biomed Pharmacother. 2017;92:1–6. 28525794 10.1016/j.biopha.2017.05.029

[35] Ducker GS, Rabinowitz JD. One-carbon metabolism in health and disease. Cell Metab. 2017;25:27–42. 27641100 10.1016/j.cmet.2016.08.009 PMC5353360

[36] Shang M, Yang H, Yang R, Chen T, Fu Y, Li Y, et al. The folate cycle enzyme MTHFD2. induces cancer immune evasion through PD-L1 up-regulation. Nat Commun. 2021;12:1940. 33782411 10.1038/s41467-021-22173-5 PMC8007798

[37] Bonagas N, Gustafsson NMS, Henriksson M, Marttila P, Gustafsson R, Wiita E, et al. Pharmacological targeting of MTHFD2 suppresses acute myeloid leukemia by inducing thymidine depletion and replication stress. Nat Cancer. 2022;3:156–72. 35228749 10.1038/s43018-022-00331-y PMC8885417

[38] Nishimura T, Nakata A, Chen X, Nishi K, Meguro-Horike M, Sasaki S, et al. Cancer stem-like properties and gefitinib resistance are dependent on purine synthetic metabolism mediated by the mitochondrial enzyme MTHFD2. Oncogene. 2019;38:2464–81. 30532069 10.1038/s41388-018-0589-1 PMC6484769

[39] Wang J, Yu Z, Jiang Y, Le T, Wu Y, Li Z, et al. Downregulation of MTHFD2 Inhibits. Proliferation and Enhances chemosensitivity in hepatocellular carcinoma via PI3K/AKT pathway. Front Biosci (Landmark Ed. 2024;29:35. 10.31083/j.fbl2901035 38287824

[40] Mo HY, Wang RB, Ma MY, Zhang Y, Li XY, Wen WR, et al. MTHFD2-mediated redox. homeostasis promotes gastric cancer progression under hypoxic conditions. Redox Rep. 2024;29:2345455. 38723197 10.1080/13510002.2024.2345455 PMC11086033

[41] Yue L, Pei Y, Zhong L, Yang H, Wang Y, Zhang W, et al. Mthfd2 modulates mitochondrial. function and dna repair to maintain the pluripotency of mouse stem cells. Stem Cell Reports. 2020;15:529–45. 32679066 10.1016/j.stemcr.2020.06.018 PMC7419720

[42] Kamerkar S, Leng C, Burenkova O, Jang SC, McCoy C, Zhang K, et al. Exosome-mediated. genetic reprogramming of tumor-associated macrophages by exoASO-STAT6 leads to potent monotherapy antitumor activity. Sci Adv. 2022;8:eabj7002. 35179953 10.1126/sciadv.abj7002 PMC8856615

[43] Alhawarri MB. Exploring the anticancer potential of furanpydone a: a computational. study on its inhibition of MTHFD2 across diverse cancer cell lines. Cell Biochem Biophys. 2024;83:437–54. 10.1007/s12013-024-01474-8 39110299

[44] Li G, Wu J, Li L, Jiang P. p53 deficiency induces MTHFD2 transcription to promote cell. proliferation and restrain DNA damage. Proc Natl Acad Sci USA. 2021;118. 10.1073/pnas.2019822118 PMC8285905 34244426

[45] Cagatay T, Ozturk M. P53 mutation as a source of aberrant beta-catenin accumulation in cancer cells. Oncogene. 2002;21:7971–80. 12439747 10.1038/sj.onc.1205919

[46] Li M, Sun D, Song N, Chen X, Zhang X, Zheng W, et al. Mutant p53 in head and neck. squamous cell carcinoma: Molecular mechanism of gain‑of‑function and targeting therapy (Review). Oncol Rep. 2023;50. 10.3892/or.2023.8599 PMC10394732 37449494

[47] Longley DB, Harkin DP, Johnston PG. 5-fluorouracil: mechanisms of action and clinical. strategies. Nat Rev Cancer. 2003;3:330–8. 12724731 10.1038/nrc1074

[48] Kim DW, Talati C, Kim R. Hepatocellular carcinoma (HCC): beyond sorafenib-chemotherapy. J Gastrointest Oncol. 2017;8:256–65. 28480065 10.21037/jgo.2016.09.07 PMC5401857

[49] Verma H, Narendra G, Raju B, Singh PK, Silakari O. Dihydropyrimidine dehydrogenase-mediated resistance to 5-fluorouracil: mechanistic investigation and solution. ACS Pharmacol Transl Sci. 2022;5:1017–33. 36407958 10.1021/acsptsci.2c00117 PMC9667542

[50] Chen JY, Chou HH, Lim SC, Huang YJ, Lai KC, Guo CL, et al. Multiomic characterization. and drug testing establish circulating tumor cells as an ex vivo tool for personalized medicine. iScience. 2022;25:105081. 36204272 10.1016/j.isci.2022.105081 PMC9529671

[51] Broutier L, Mastrogiovanni G, Verstegen MM, Francies HE, Gavarro LM, Bradshaw CR, et al. Human primary liver cancer-derived organoid cultures for disease modeling and drug screening. Nat Med. 2017;23:1424–35. 29131160 10.1038/nm.4438 PMC5722201

[52] Lin RJ, Kuo MW, Yang BC, Tsai HH, Chen K, Huang JR, et al. B3GALT5 knockout alters gycosphingolipid profile and facilitates transition to human naive pluripotency. Proc Natl Acad Sci USA. 2020;117:27435–44. 33087559 10.1073/pnas.2003155117 PMC7959494

[53] Lin CY, Wu RC, Huang CY, Lai CH, Chao AS, Li HP, et al. A patient-derived xenograft. model of dedifferentiated endometrial carcinoma: a proof-of-concept study for the identification of new molecularly informed treatment approaches. Cancers (Basel). 2021;13. 10.3390/cancers13235962 PMC8656552 34885073

[54] Tsai CN, Yu MC, Lee YS, Feng KC, Wu CH, Li YC, et al. NRF2-SOX4 complex regulates. PSPH in hepatocellular carcinoma and modulates M2 macrophage differentiation. Cancer Gene Ther. 2025. 10.1038/s41417-025-00951-3 40855366

[55] Lin CY, Wu KY, Chi LM, Tang YH, Huang HJ, Lai CH, et al. Starvation-inactivated MTOR. triggers cell migration via a ULK1-SH3PXD2A/TKS5-MMP14 pathway in ovarian carcinoma. Autophagy. 2023;19:3151–68. 37505094 10.1080/15548627.2023.2239633 PMC10621272

[56] Ianevski A, Giri AK, Aittokallio T. SynergyFinder 2.0: visual analytics of multi-drug combination synergies. Nucleic Acids Res. 2020;48:W488–93. 32246720 10.1093/nar/gkaa216 PMC7319457

[57] Li HP, Huang CY, Lui KW, Chao YK, Yeh CN, Lee LY, et al. Combination of epithelial. growth factor receptor blockers and CDK4/6 inhibitor for nasopharyngeal carcinoma treatment. Cancers (Basel). 2021;13. 10.3390/cancers13122954 PMC8231497 34204797

[58] Ma H, Zhang J, Zhou L, Wen S, Tang HY, Jiang B, et al. c-Src promotes tumorigenesis and. tumor progression by activating PFKFB3. Cell Rep. 2020;30:4235–49 e6. 32209481 10.1016/j.celrep.2020.03.005

[59] Pang Z, Lu Y, Zhou G, Hui F, Xu L, Viau C, et al. MetaboAnalyst 6.0: towards a unified. platform for metabolomics data processing, analysis and interpretation. Nucleic Acids Res. 2024;52:W398–406. 38587201 10.1093/nar/gkae253 PMC11223798

[60] Zhu Z, Leung GKK. More than a metabolic enzyme: MTHFD2 as a novel target for. anticancer therapy? Front Oncol. 2020;10:658. 32411609 10.3389/fonc.2020.00658 PMC7199629

