# Supplementary material for: SOX4-STAT6-MTHFD2 axis drives hepatocellular carcinoma progression and treatment resistance
Source: Cell Death Dis. 2026 Jan 3;17(1):154. doi: 10.1038/s41419-025-08394-2 (PMC12858837; doi:10.1038/s41419-025-08394-2)
Supplement: Supplementary file 1 — Supplementary Figures and Tables [file 41419_2025_8394_MOESM1_ESM.docx]

**SOX4-STAT6-MTHFD2 Axis Drives Hepatocellular Carcinoma Progression and Treatment Resistance**

Chia-Lung Tsai^#^, Ming-Chin Yu^#^, Cheng-Lung Hsu, Hsiang-Yu Tang, Yun-Shien Lee, Lang-Ming Chi, Sey-En Lin, Mei-Ling Cheng, Heng-Yuan Hsu, Chi-Neu Tsai^*^

**Supplementary Figure and Tables**

**Supplementary Fig. 1** Protein expression of SOX4, STAT6, and MTHFD2 were analyzed by western blotting in hepatocytes, HCC cell lines and a hepatoblastoma cell line

**Supplementary Fig. 2** The genes selected from SOX4 or STAT6 ChIP versus RNA seq. results in this study.

**Supplementary Fig. 3** Protein expression of SOX4, STAT6, p-STAT6Y641 and MTHFD2 in clinical specimen of HCC.

**Supplementary Fig. 4** Expression of SOX4, STAT6, and MTHFD2 in HCC specimens assessed by immunohistochemistry (IHC)

**Supplementary Fig. 5** The metabolite profile in SOX4/STAT6/MTHFD2^high^ and SOX4/STAT6/MTHFD2^low^ HCC tumor lesions (*n*=21) was analyzed by a LC-MS/MS

**Supplementary Fig. 6** Expression of SOX4, STAT6, MTHFD2 and corelated with response to Sorafenib using transcript data from GSE109211.

**Supplementary Fig. 7** The effect of DS18561882 & Sorafenib, AS1517499 & Sorafenib in Hep3B and SNU-475 cells as revealed by CCK8 assay following calculating by Zero interaction potency (ZIP) model using SynergyFinder 3.0 website

**Supplementary** Fig**.** **8** The HCC PDx model used in this study

**Supplementary Fig. 9** The effect of DS18561882 & 5-FU, AS1517499 & 5-FU in Hep3B and SNU-475 cells as revealed by CCK8 assay following calculating by ZIP synergy model using SynergyFinder 3.0 website.

**Supplementary Table 1 The genes regulated by SOX4 and STAT6 in Hep3B cells**

**Supplementary Table 2.** Demographic data of enrolled patients with HCC in this study

**Supplementary Table 3.** The information of cell lines used in this study

**Supplementary Table 4.** Antibodies used in this study

**Supplementary Table 5** Primers used in this study

**Reference**


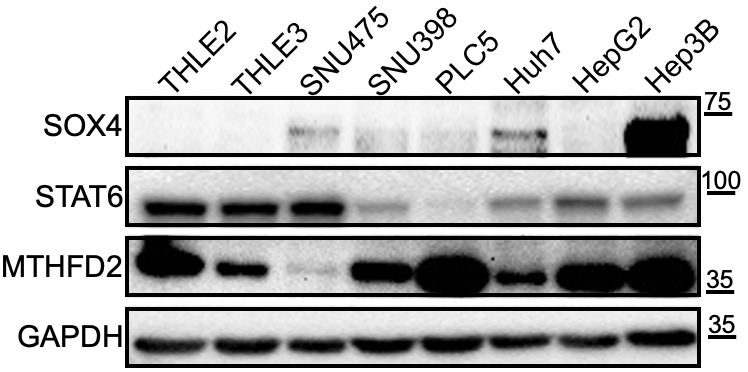


**Supplementary Fig. 1 Protein expression of SOX4, STAT6, and MTHFD2 were analyzed by western blotting in hepatocytes, HCC cell lines and a hepatoblastoma cell line.** The protein expression of SOX4, total STAT6 and MTHFD2 were analyzed in two immortalized hepatocytes (THLE2, THLE3), five HCC cell lines and one hepatoblastoma cell (HepG2) using western blotting. GAPDH was used as an internal control for protein quantification & normalization.


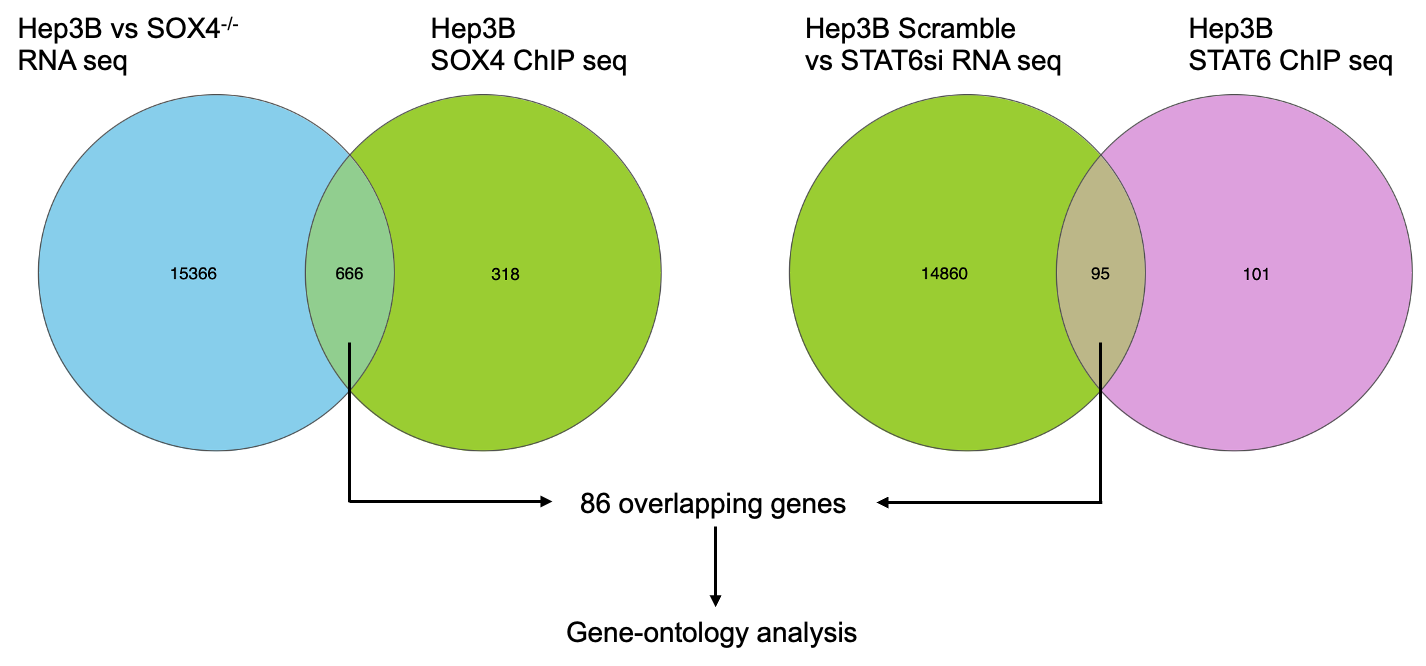


**Supplementary Fig. 2** **The genes selected from SOX4 or STAT6 ChIP versus RNA seq. results in this study.** The selection criteria for the genes co-regulated by SOX4 versus STAT6 in Hep3B cells as shown by Venna diagram. The SOX4 ChIP seq. results were integrated with the differential gene expression profile from Hep3B vs. Hep3B SOX4^-/-^ cells, whereas the STAT6 ChIP results were also integrated with differential gene expression profile from Hep3B transfected with Scramble vs. STAT6 siRNA. Finally, 666 genes and 95 genes could be selected from SOX4 and STAT6 ChIP/RNA seq. results (GSE277540), respectively. The 86 overlapping genes from SOX4 and STAT6 ChIP/RNA seq. results were analyzed using Gene Ontology (GO) enrichment analysis via Metascape (http://metascape.org).


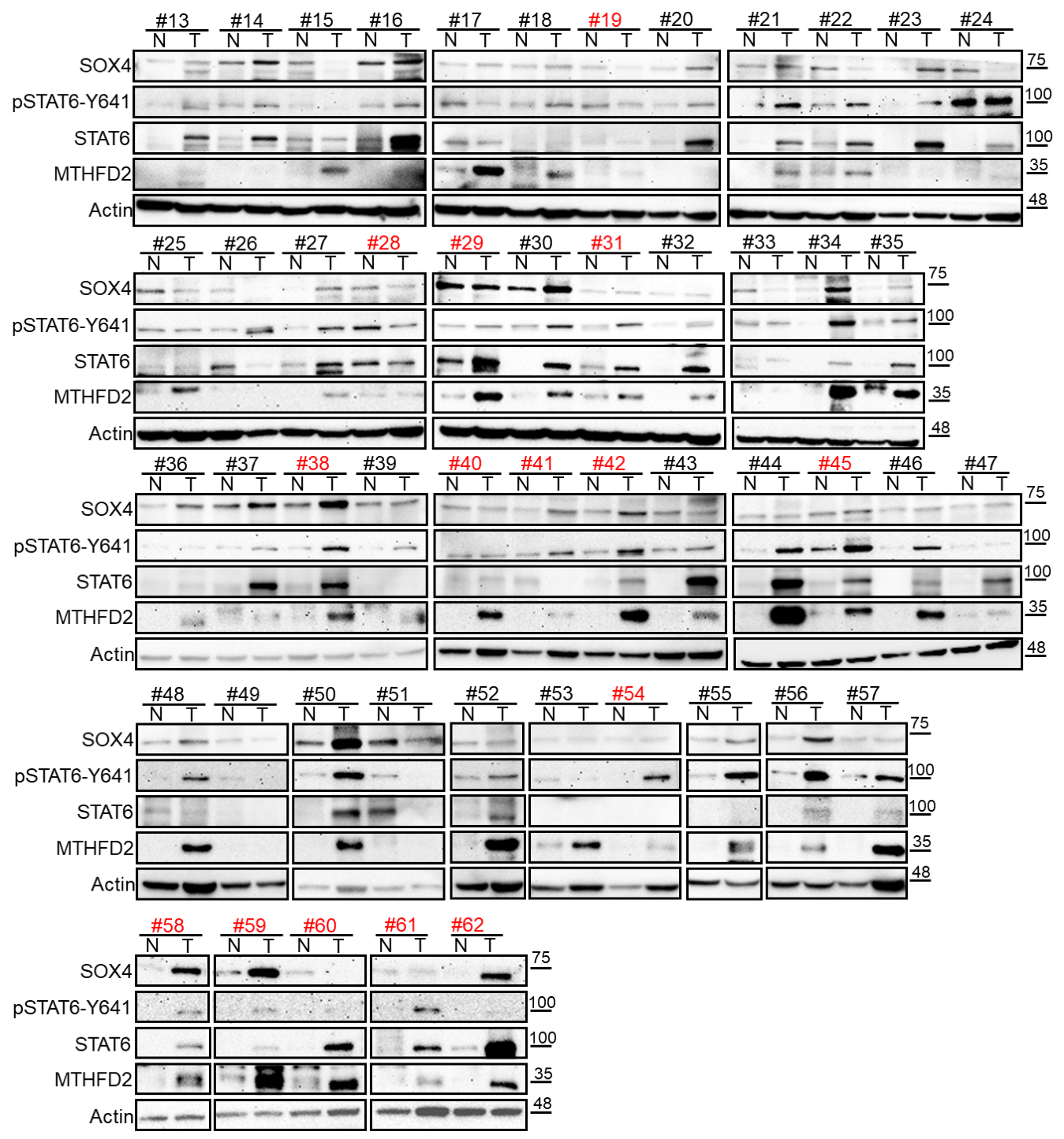


**Supplementary Fig. 3 Protein expression of SOX4, STAT6, p-STAT6Y641 and MTHFD2 in clinical specimen of HCC.** The protein expression levels of SOX4, p-STAT6 (Y641), total STAT6, and MTHFD2 were analyzed in normal adjacent (N) versus tumor (T) tissues from our enrolled clinical specimens of HCC (*n*=58) using western blotting. β-actin (Actin) was used as an internal control for ensure the accuracy of the protein quantification and normalization. The patients who received TKI, or immunotherapy, or combined treatment are shown as red color in their enrolled number.


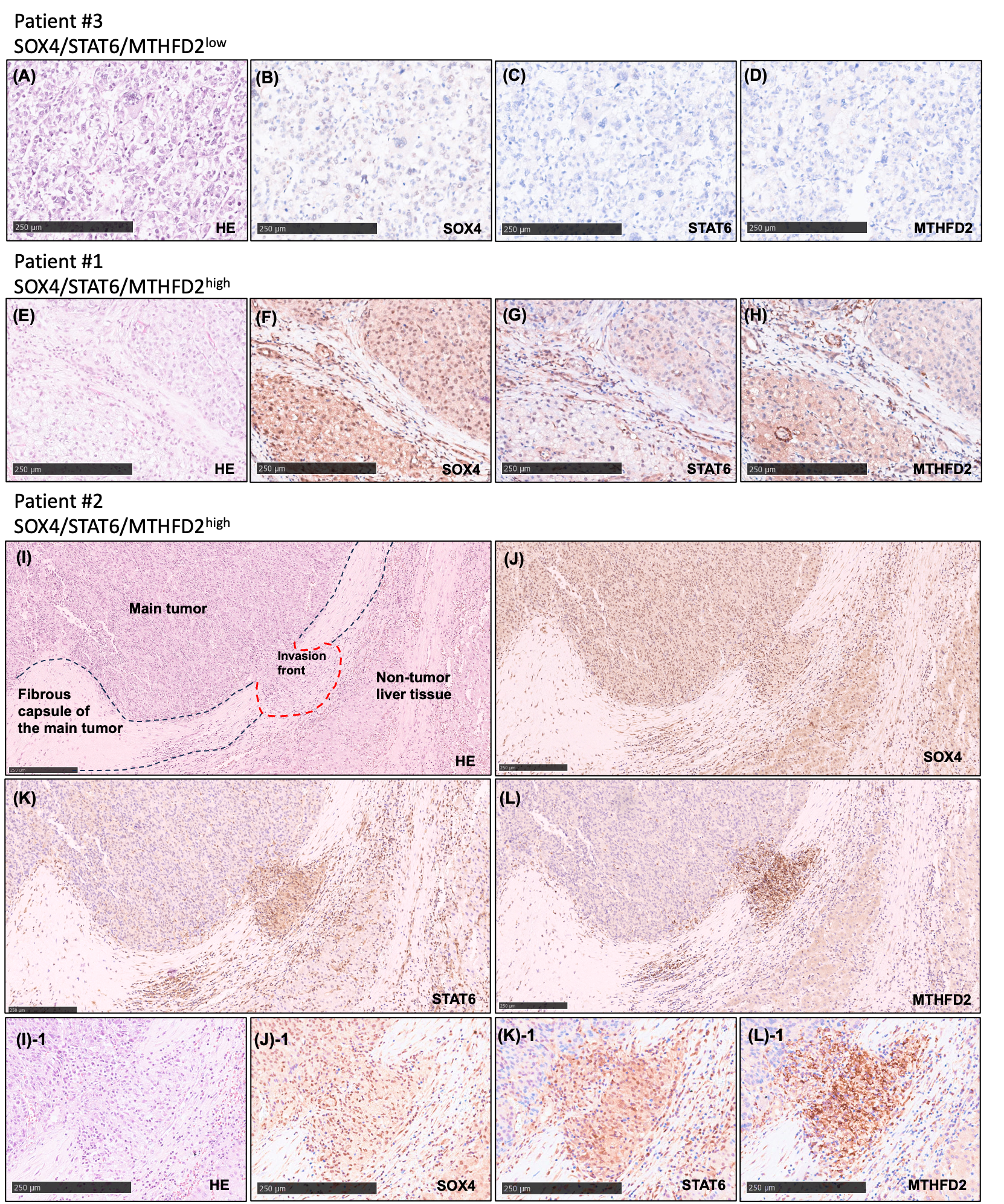


**Supplementary Fig. 4 Expression of SOX4, STAT6, and MTHFD2 in HCC specimens assessed by immunohistochemistry (IHC)**. (A–D) In patient #3, Western blotting (Fig. 6A) revealed low expression of SOX4, STAT6, and MTHFD2. Corresponding FFPE sections were examined by H&E and IHC staining for SOX4, STAT6, and MTHFD2. (E–H) In patient #1, Western blotting (Fig. 6A) showed high expression of SOX4, STAT6, and MTHFD2. FFPE sections were analyzed accordingly by H&E and IHC staining.
(I–L) In patient #2, Western blotting (Fig. 6A) indicated high overall expression of SOX4, STAT6, and MTHFD2. The tumor invasion front was visible in H&E staining as a tongue-like cluster of HCC cells invading the fibrous capsule [I, (I)-1]. Cells at the invasion front displayed stronger IHC intensities of SOX4 [J, (J)-1], STAT6 [K, (K)-1], and MTHFD2 [L, (L)-1]. Scale bar: 250 μm.


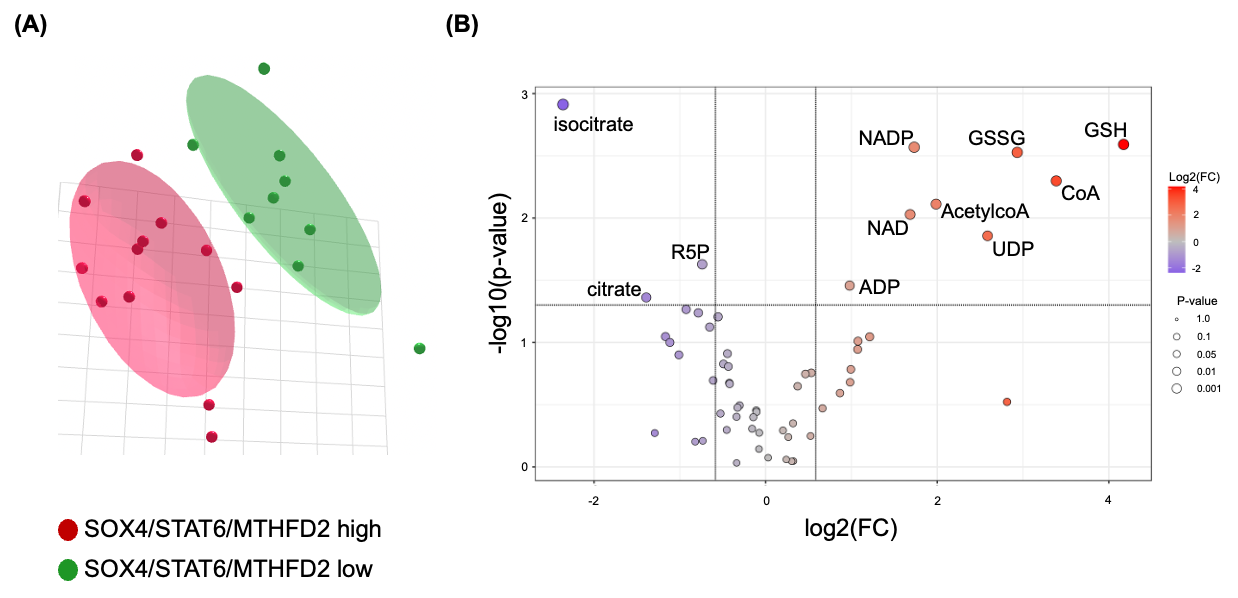


**Supplementary Fig. 5 The metabolite profile in SOX4/STAT6/MTHFD2^high^ and SOX4/STAT6/MTHFD2^low^ HCC tumor lesions (*n*=21) was analyzed by a LC-MS/MS.** The expression of SOX4 was classified as SOX4/STAT6/MTHFD2^high^ and SOX4/STAT6/MTHFD2^low^ based on its western blotting results (Supplementary Fig. 5), then the metabolites of each sample were analyzed by LC-MS/MS. The metabolite results were further analyzed by MetaboAnalyst 6.0 website (https://www.metaboanalyst.ca/home.xhtml) [1]. (A). The Metabolomic profiles of SOX4/STAT6/MTHFD2^high^ and SOX4/STAT6/MTHFD2^low^ group were analyzed using partial least squares–discriminate analysis (PLS–DA) plot. (B). The most significant up-regulated (red color) and down-regulated metabolites between SOX4/STAT6/MTHFD2^high^ and SOX4/STAT6/MTHFD2^low^ group is shown by volcano scatter plot.

**
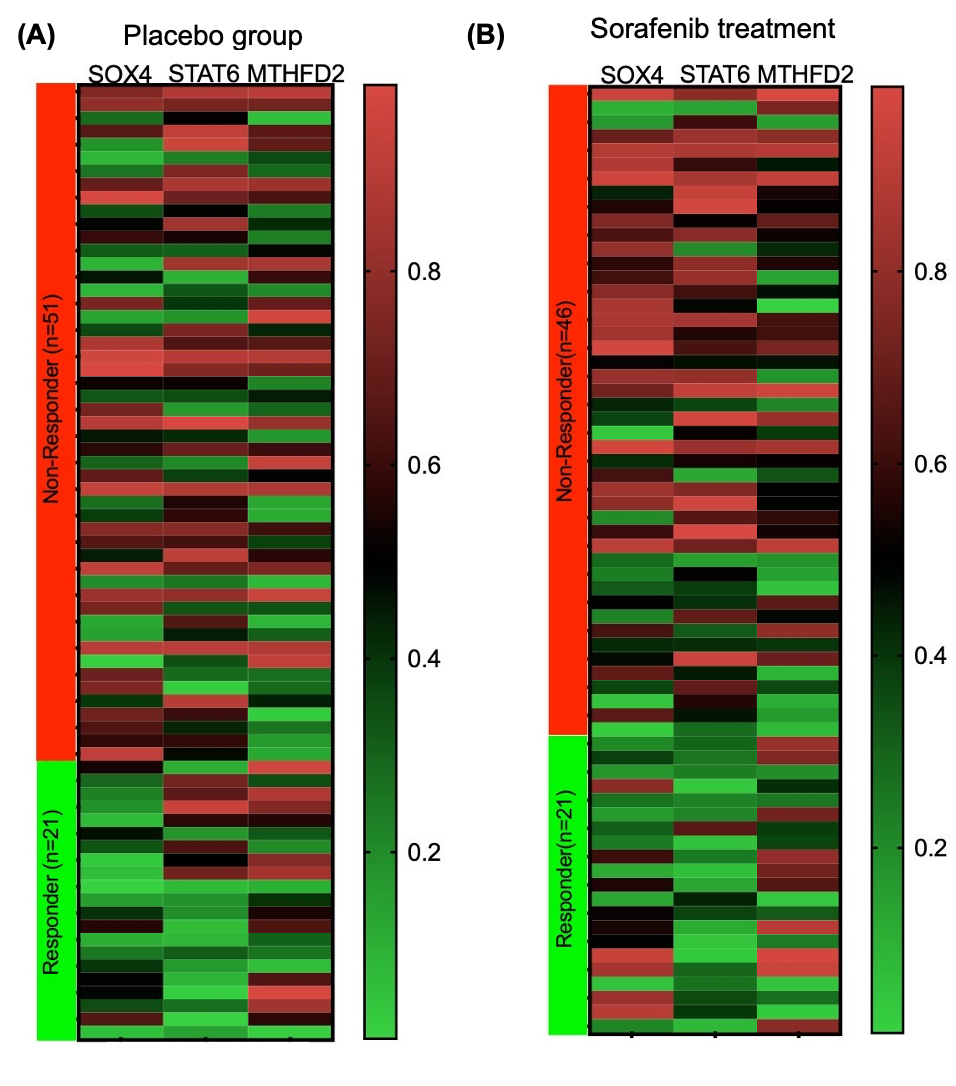
**

**
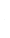

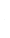

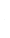
Supplementary Fig. 6 Expression of SOX4, STAT6, and MTHFD2 in relation to Sorafenib response using transcriptomic data from GSE109211[2].** Transcript levels of SOX4, STAT6, and MTHFD2 in tumor lesions were normalized and displayed as percentile gene expression (green to red, 0 to 1 scale bar). (A) Placebo group. (B) Sorafenib-treated group. The numbers of responders and non-responders in each group are indicated.

**
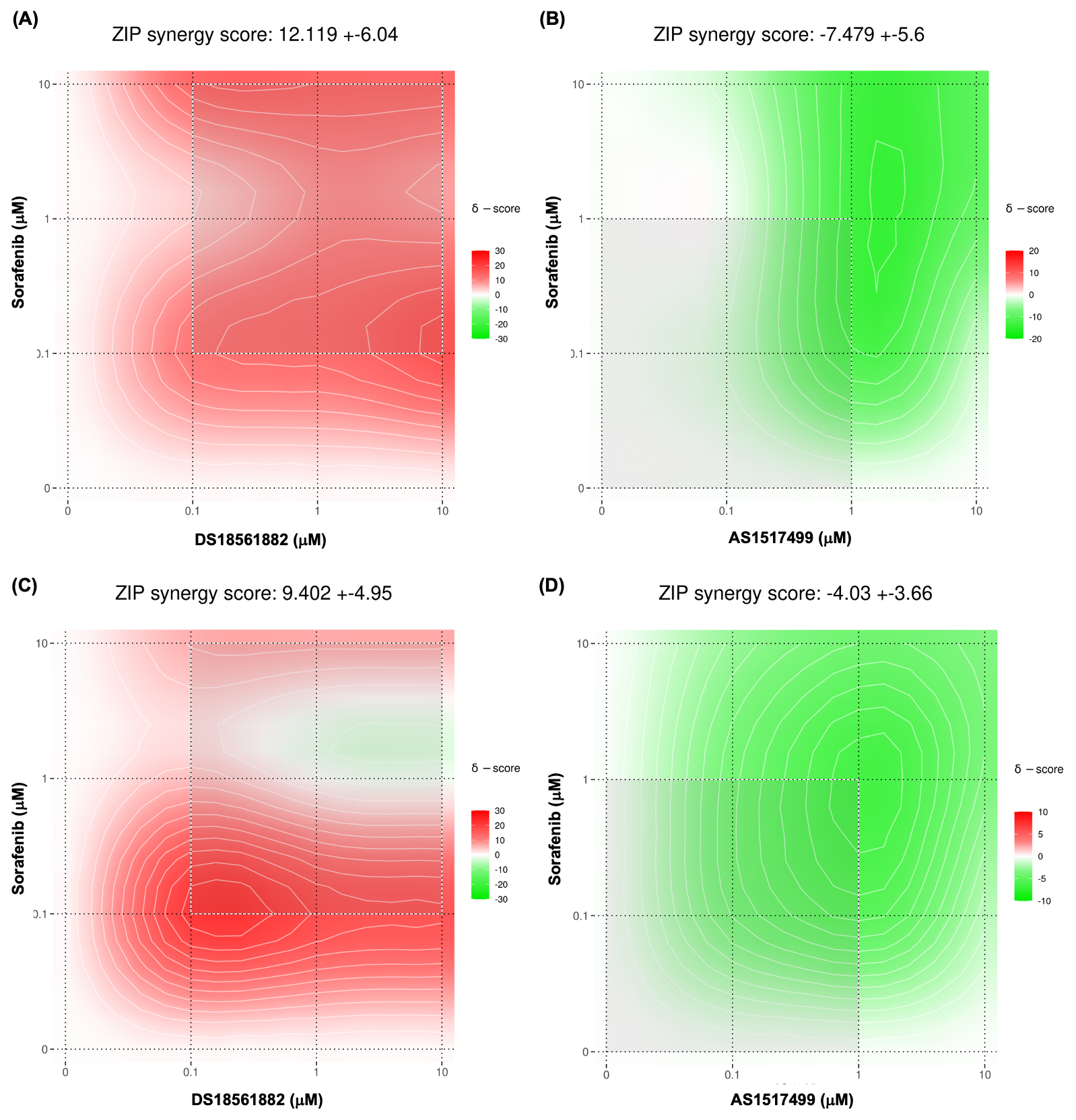
**

**Supplementary Fig. 7** The effect of DS18561882 & Sorafenib, AS1517499 & Sorafenib in Hep3B and SNU-475 cells as revealed by CCK8 assay following calculation by Zero interaction potency (ZIP) model using SynergyFinder 3.0 website [3]. (A)(B). Hep3B cells were treated with DS18561882 & Sorafenib (A), or AS1517499 & Sorafenib (B) at 0, 0.1, 1, and 10 μM for 24 hours then cell viability was measured by CCK8 assay. (C)(D). SNU475 cells were treated with DS18561882 & Sorafenib (A), or AS1517499 & Sorafenib (B) at 0, 0.1, 1, and 10 μM for 24 hours then cell viability was measured by CCK8 assay. At least three independent assays of CCK8 results in each cell line were applied into SynergyFinder 3.0 website to calculate ZIP synergy score. The interaction of two drugs is defined as antagonistic, additive or synergistic if the ZIP score is less than -10, -10 to 10, larger than 10; respectively [3].


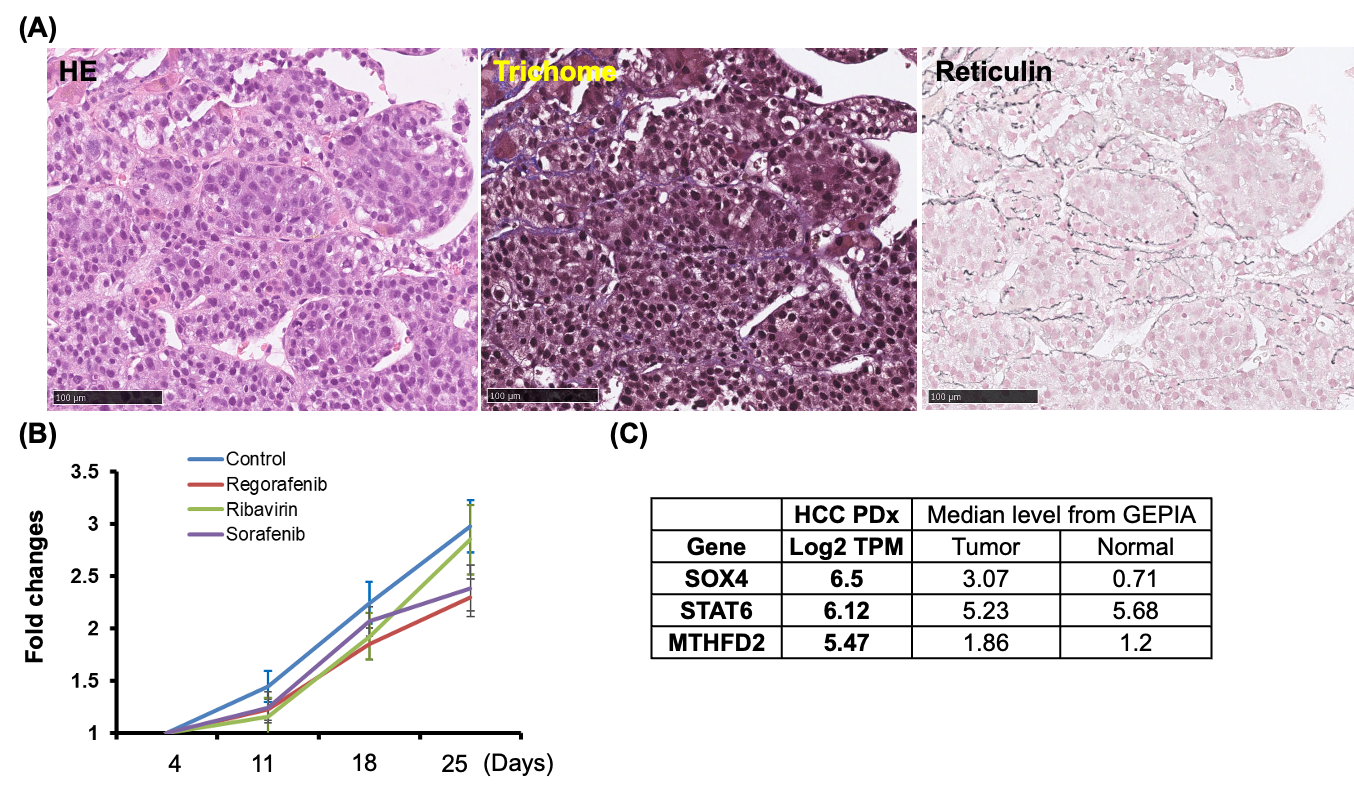


**Supplementary Fig. 8 The HCC PDx model used in this study** (A). The hematoxylin and eosin (HE), trichome, and reticulum staining of a patient with HBV+ HCC. (B). The tumors were cut and implanted to 5-week-old, anesthetized NPG (NOD.Cg-Prkdcscid Il2rgtm1Vst/Vst) mice subcutaneously, the inhibitors were administered 4 weeks after tumor transplantation in mice. There were three mice in either control or each treatment group, the tumor volume was measured as length x width x height (mm3) as X axis showed. (C). The transcriptome of this HBV+ HCC tumor was analyzed by RNA seq., the transcripts of SOX4, STAT6, MTHFD2 in tumor lesions are shown as the log2 TPM.

**
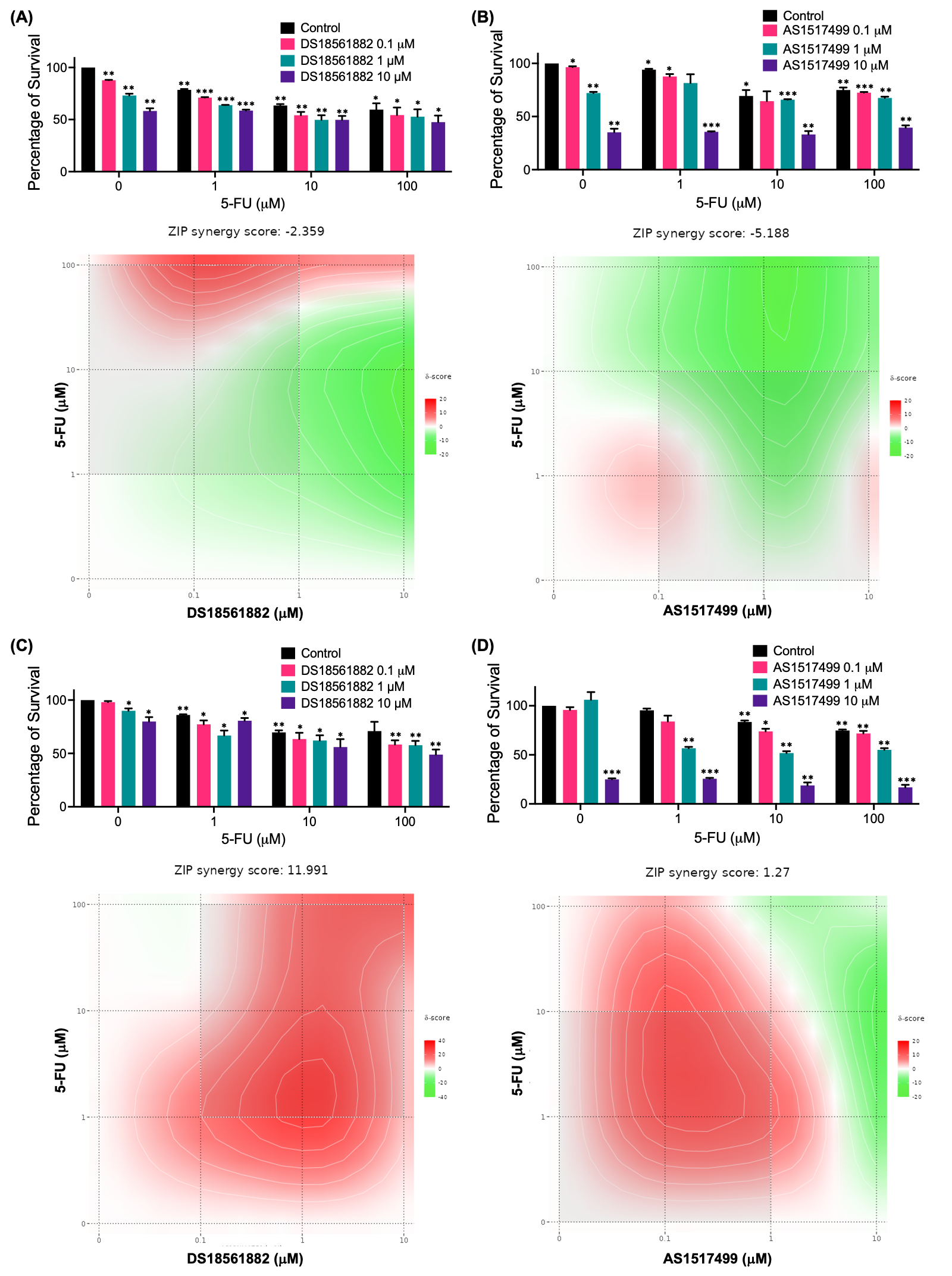
**

**Supplementary Fig. 9** The effect of DS18561882 & 5-FU, AS1517499 & 5-FU in Hep3B and SNU-475 cells as revealed by CCK8 assay following calculation using ZIP synergy model using the SynergyFinder 3.0 website [3]. (A)(B). Hep3B cells were treated with DS18561882 & 5-FU (A), or AS1517499 & 5-FU (B) at 0, 0.1, 1, and 10 μM for 24 hours then cell viability was measured by CCK8 assay (upper panel). The ZIP synergy score of each treatment is shown at the lower panel. (C)(D). SNU475 cells were treated with DS18561882 & 5-FU (C), or AS1517499 & 5-FU (D) at 0, 0.1, 1, and 10 μM for 24 hours then cell viability was measured by CCK8 assay (upper panel). The ZIP synergy score of each treatment is shown at lower panel. At least three independent assays of CCK8 results in each cell line were applied into SynergyFinder 3.0 website to calculate the ZIP synergy score. The interaction of two drugs is defined as antagonistic, additive or synergistic as per the ZIP score being less than -10, -10 to 10, larger than 10; respectively [3].

| **Supplementary Table 1** The genes regulated by SOX4 and STAT6 in Hep3B cells | | |
| --- | --- | --- |
| **Gene Symbol** | **Description** | **Biological Process (GO)** |
| ABCB1 | ATP binding cassette subfamily B member 1 | GO:0099040 ceramide translocation;GO:0046864 isoprenoid transport;GO:0046865 terpenoid transport |
| ABCB4 | ATP binding cassette subfamily B member 4 | GO:1901557 response to fenofibrate;GO:1903413 cellular response to bile acid;GO:0032782 bile acid secretion |
| ABCG2 | ATP binding cassette subfamily G member 2 (JR blood group) | GO:0015878 biotin transport;GO:0097744 renal urate salt excretion;GO:0032218 riboflavin transport |
| ACE | angiotensin I converting enzyme | GO:0002019 regulation of renal output by angiotensin;GO:0060177 regulation of angiotensin metabolic process;GO:0071838 cell proliferation in bone marrow |
| ADRB1 | adrenoceptor beta 1 | GO:0001997 positive regulation of the force of heart contraction by epinephrine-norepinephrine;GO:0001996 positive regulation of heart rate by epinephrine-norepinephrine;GO:0003099 positive regulation of the force of heart contraction by chemical signal |
| ASIC1 | acid sensing ion channel subunit 1 | GO:0046929 negative regulation of neurotransmitter secretion;GO:0051589 negative regulation of neurotransmitter transport;GO:0050915 sensory perception of sour taste |
| ASIC3 | acid sensing ion channel subunit 3 | GO:0042930 enterobactin transport;GO:0015891 siderophore transport;GO:0050968 detection of chemical stimulus involved in sensory perception of pain |
| BCL2 | BCL2 apoptosis regulator | GO:0032848 negative regulation of cellular pH reduction;GO:0043375 CD8-positive, alpha-beta T cell lineage commitment;GO:0046671 negative regulation of retinal cell programmed cell death |
| CHPF | chondroitin polymerizing factor | GO:0050653 chondroitin sulfate proteoglycan biosynthetic process, polysaccharide chain biosynthetic process;GO:0030206 chondroitin sulfate biosynthetic process;GO:0050650 chondroitin sulfate proteoglycan biosynthetic process |
| CLCN6 | chloride voltage-gated channel 6 | GO:0006884 cell volume homeostasis;GO:1902476 chloride transmembrane transport;GO:0006821 chloride transport |
| COG1 | component of oligomeric golgi complex 1 | GO:0000301 retrograde transport, vesicle recycling within Golgi;GO:0006891 intra-Golgi vesicle-mediated transport;GO:0007030 Golgi organization |
| COL18A1 | collagen type XVIII alpha 1 chain | GO:0051599 response to hydrostatic pressure;GO:0001886 endothelial cell morphogenesis;GO:0009415 response to water |
| COMT | catechol-O-methyltransferase | GO:0048243 norepinephrine secretion;GO:0016036 cellular response to phosphate starvation;GO:0001999 renal response to blood flow involved in circulatory renin-angiotensin regulation of systemic arterial blood pressure |
| CYP1A1 | cytochrome P450 family 1 subfamily A member 1 | GO:0009692 ethylene metabolic process;GO:0017143 insecticide metabolic process;GO:0019341 dibenzo-p-dioxin catabolic process |
| CYP1B1 | cytochrome P450 family 1 subfamily B member 1 | GO:0002930 trabecular meshwork development;GO:0071387 cellular response to cortisol stimulus;GO:1904681 response to 3-methylcholanthrene |
| CYP2B6 | cytochrome P450 family 2 subfamily B member 6 | GO:0019373 epoxygenase P450 pathway;GO:0042178 xenobiotic catabolic process;GO:0019369 arachidonate metabolic process |
| CYP2C18 | cytochrome P450 family 2 subfamily C member 18 | GO:0043651 linoleic acid metabolic process;GO:0042573 retinoic acid metabolic process;GO:0042572 retinol metabolic process |
| CYP2J2 | cytochrome P450 family 2 subfamily J member 2 | GO:0019373 epoxygenase P450 pathway;GO:0043651 linoleic acid metabolic process;GO:0019369 arachidonate metabolic process |
| CYP2R1 | cytochrome P450 family 2 subfamily R member 1 | GO:0010164 response to cesium ion;GO:0036378 calcitriol biosynthetic process from calciol;GO:0042368 vitamin D biosynthetic process |
| CYP2S1 | cytochrome P450 family 2 subfamily S member 1 | GO:1903604 cytochrome metabolic process;GO:0019373 epoxygenase P450 pathway;GO:0042573 retinoic acid metabolic process |
| CYP2W1 | cytochrome P450 family 2 subfamily W member 1 | GO:0016103 diterpenoid catabolic process;GO:0034653 retinoic acid catabolic process;GO:1903604 cytochrome metabolic process |
| CYP3A7 | cytochrome P450 family 3 subfamily A member 7 | GO:0002933 lipid hydroxylation;GO:0070989 oxidative demethylation;GO:0070988 demethylation |
| CYP3A7-CYP3A51P | CYP3A7-CYP3A51P readthrough |  |
| DPYD | dihydropyrimidine dehydrogenase | GO:0006214 thymidine catabolic process;GO:0046127 pyrimidine deoxyribonucleoside catabolic process;GO:0006212 uracil catabolic process |
| EFCAB5 | EF-hand calcium binding domain 5 |  |
| ENOSF1 | enolase superfamily member 1 | GO:0009063 amino acid catabolic process;GO:0016052 carbohydrate catabolic process;GO:0006520 amino acid metabolic process |
| F5 | coagulation factor V | GO:0032571 response to vitamin K;GO:0033273 response to vitamin;GO:0007584 response to nutrient |
| FGA | fibrinogen alpha chain | GO:2000258 negative regulation of protein activation cascade;GO:2000260 regulation of blood coagulation, common pathway;GO:2000261 negative regulation of blood coagulation, common pathway |
| G6PD | glucose-6-phosphate dehydrogenase | GO:0010732 regulation of protein glutathionylation;GO:0010734 negative regulation of protein glutathionylation;GO:0019322 pentose biosynthetic process |
| GPR68 | G protein-coupled receptor 68 | GO:2001206 positive regulation of osteoclast development;GO:0045656 negative regulation of monocyte differentiation;GO:0036035 osteoclast development |
| HCG4B | HLA complex group 4B |  |
| HCP5 | HLA complex P5 |  |
| HLA-A | major histocompatibility complex, class I, A | GO:0002485 antigen processing and presentation of endogenous peptide antigen via MHC class I via ER pathway, TAP-dependent;GO:2000567 regulation of memory T cell activation;GO:2000568 positive regulation of memory T cell activation |
| HLA-C | major histocompatibility complex, class I, C | GO:0002486 antigen processing and presentation of endogenous peptide antigen via MHC class I via ER pathway, TAP-independent;GO:0002476 antigen processing and presentation of endogenous peptide antigen via MHC class Ib;GO:0002484 antigen processing and presentation of endogenous peptide antigen via MHC class I via ER pathway |
| HLA-E | major histocompatibility complex, class I, E | GO:0001815 positive regulation of antibody-dependent cellular cytotoxicity;GO:0001813 regulation of antibody-dependent cellular cytotoxicity;GO:0032679 regulation of TRAIL production |
| HLA-F | major histocompatibility complex, class I, F | GO:0043322 negative regulation of natural killer cell degranulation;GO:0002728 negative regulation of natural killer cell cytokine production;GO:0002477 antigen processing and presentation of exogenous peptide antigen via MHC class Ib |
| HLA-F-AS1 | HLA-F antisense RNA 1 |  |
| HLA-H | major histocompatibility complex, class I, H (pseudogene) | GO:0002486 antigen processing and presentation of endogenous peptide antigen via MHC class I via ER pathway, TAP-independent;GO:0002476 antigen processing and presentation of endogenous peptide antigen via MHC class Ib;GO:0002484 antigen processing and presentation of endogenous peptide antigen via MHC class I via ER pathway |
| HLA-J | major histocompatibility complex, class I, J (pseudogene) |  |
| HLA-L | major histocompatibility complex, class I, L (pseudogene) |  |
| HNRNPA1 | heterogeneous nuclear ribonucleoprotein A1 | GO:1903936 cellular response to sodium arsenite;GO:1902075 cellular response to salt;GO:1903935 response to sodium arsenite |
| HNRNPA1L2 | heterogeneous nuclear ribonucleoprotein A1 like 2 | GO:0051028 mRNA transport;GO:0050657 nucleic acid transport;GO:0050658 RNA transport |
| KCNK1 | potassium two pore domain channel subfamily K member 1 | GO:0060075 regulation of resting membrane potential;GO:0014047 glutamate secretion;GO:0071468 cellular response to acidic pH |
| KDM1B | lysine demethylase 1B | GO:0044726 epigenetic programing of female pronucleus;GO:0044725 epigenetic programming in the zygotic pronuclei;GO:0071514 genomic imprinting |
| LARS2 | leucyl-tRNA synthetase 2, mitochondrial | GO:0006429 leucyl-tRNA aminoacylation;GO:0106074 aminoacyl-tRNA metabolism involved in translational fidelity;GO:0006450 regulation of translational fidelity |
| LECT2 | leukocyte cell derived chemotaxin 2 | GO:0006935 chemotaxis;GO:0042330 taxis;GO:0040011 locomotion |
| MICA | MHC class I polypeptide-related sequence A | GO:0002418 immune response to tumor cell;GO:0032815 negative regulation of natural killer cell activation;GO:0001913 T cell mediated cytotoxicity |
| MICB | MHC class I polypeptide-related sequence B | GO:0050689 negative regulation of defense response to virus by host;GO:0050687 negative regulation of defense response to virus;GO:0046629 gamma-delta T cell activation |
| MTHFD2 | methylenetetrahydrofolate dehydrogenase (NADP+ dependent) 2, methenyltetrahydrofolate cyclohydrolase | GO:0035999 tetrahydrofolate interconversion;GO:0046655 folic acid metabolic process;GO:0046653 tetrahydrofolate metabolic process |
| MTHFR | methylenetetrahydrofolate reductase | GO:0033274 response to vitamin B2;GO:0035999 tetrahydrofolate interconversion;GO:0046500 S-adenosylmethionine metabolic process |
| MXD1 | MAX dimerization protein 1 | GO:0000122 negative regulation of transcription by RNA polymerase II;GO:0045892 negative regulation of DNA-templated transcription;GO:1902679 negative regulation of RNA biosynthetic process |
| NAT1 | N-acetyltransferase 1 | GO:0006805 xenobiotic metabolic process;GO:0071466 cellular response to xenobiotic stimulus;GO:0009410 response to xenobiotic stimulus |
| NAT2 | N-acetyltransferase 2 | GO:0006805 xenobiotic metabolic process;GO:0071466 cellular response to xenobiotic stimulus;GO:0009410 response to xenobiotic stimulus |
| NLK | nemo like kinase | GO:0007223 Wnt signaling pathway, calcium modulating pathway;GO:0018107 peptidyl-threonine phosphorylation;GO:0071470 cellular response to osmotic stress |
| NUDT15 | nudix hydrolase 15 | GO:0006203 dGTP catabolic process;GO:0046070 dGTP metabolic process;GO:0009217 purine deoxyribonucleoside triphosphate catabolic process |
| OSBPL10 | oxysterol binding protein like 10 | GO:0036150 phosphatidylserine acyl-chain remodeling;GO:0006658 phosphatidylserine metabolic process;GO:0015914 phospholipid transport |
| PPP2R2C | protein phosphatase 2 regulatory subunit Bgamma |  |
| PRICKLE1 | prickle planar cell polarity protein 1 | GO:0061864 basement membrane constituent secretion;GO:0061865 polarized secretion of basement membrane proteins in epithelium;GO:1905070 anterior visceral endoderm cell migration |
| PRKG1 | protein kinase cGMP-dependent 1 | GO:0010920 negative regulation of inositol phosphate biosynthetic process;GO:0014050 negative regulation of glutamate secretion;GO:0060087 relaxation of vascular associated smooth muscle |
| PYM1 | PYM homolog 1, exon junction complex associated factor | GO:1903259 exon-exon junction complex disassembly;GO:0000184 nuclear-transcribed mRNA catabolic process, nonsense-mediated decay;GO:0000956 nuclear-transcribed mRNA catabolic process |
| REP15 | RAB15 effector protein | GO:0033572 transferrin transport;GO:0001881 receptor recycling;GO:0043112 receptor metabolic process |
| RUNX2 | RUNX family transcription factor 2 | GO:0036076 ligamentous ossification;GO:0002051 osteoblast fate commitment;GO:1904383 response to sodium phosphate |
| SEMA3A | semaphorin 3A | GO:0150018 basal dendrite development;GO:0150019 basal dendrite morphogenesis;GO:0150020 basal dendrite arborization |
| SFT2D1 | SFT2 domain containing 1 | GO:0015031 protein transport;GO:0016192 vesicle-mediated transport;GO:0045184 establishment of protein localization |
| SLC19A1 | solute carrier family 19 member 1 | GO:0051958 methotrexate transport;GO:1904447 folate import across plasma membrane;GO:0098838 folate transmembrane transport |
| SLC22A25 | solute carrier family 22 member 25 | GO:0015711 organic anion transport;GO:0055085 transmembrane transport;GO:0006810 transport |
| SLCO1B1 | solute carrier organic anion transporter family member 1B1 | GO:0042167 heme catabolic process;GO:0046149 pigment catabolic process;GO:0070327 thyroid hormone transport |
| SLCO1B3 | solute carrier organic anion transporter family member 1B3 | GO:0042167 heme catabolic process;GO:0046149 pigment catabolic process;GO:0006787 porphyrin-containing compound catabolic process |
| SMG1 | SMG1 nonsense mediated mRNA decay associated PI3K related kinase | GO:0000184 nuclear-transcribed mRNA catabolic process, nonsense-mediated decay;GO:0046854 phosphatidylinositol phosphate biosynthetic process;GO:0006406 mRNA export from nucleus |
| SMURF1 | SMAD specific E3 ubiquitin protein ligase 1 | GO:0061736 engulfment of target by autophagosome;GO:0061753 substrate localization to autophagosome;GO:0071211 protein targeting to vacuole involved in autophagy |
| SULT1A1 | sulfotransferase family 1A member 1 | GO:0009812 flavonoid metabolic process;GO:0006068 ethanol catabolic process;GO:0006067 ethanol metabolic process |
| SULT1A2 | sulfotransferase family 1A member 2 | GO:0006068 ethanol catabolic process;GO:0006067 ethanol metabolic process;GO:0034310 primary alcohol catabolic process |
| SULT1A3 | sulfotransferase family 1A member 3 | GO:0019614 catechol-containing compound catabolic process;GO:0042420 dopamine catabolic process;GO:0042424 catecholamine catabolic process |
| THSD1 | thrombospondin type 1 domain containing 1 | GO:0048041 focal adhesion assembly;GO:0007044 cell-substrate junction assembly;GO:0150115 cell-substrate junction organization |
| TMEM143 | transmembrane protein 143 | GO:0008150 biological_process |
| TPMT | thiopurine S-methyltransferase | GO:0042178 xenobiotic catabolic process;GO:0006805 xenobiotic metabolic process;GO:0071466 cellular response to xenobiotic stimulus |
| TRAK1 | trafficking kinesin protein 1 | GO:0098957 anterograde axonal transport of mitochondrion;GO:0019896 axonal transport of mitochondrion;GO:0048311 mitochondrion distribution |
| TRPV1 | transient receptor potential cation channel subfamily V member 1 | GO:1901594 response to capsazepine;GO:0090212 negative regulation of establishment of blood-brain barrier;GO:0001660 fever generation |
| TRPV4 | transient receptor potential cation channel subfamily V member 4 | GO:0097497 blood vessel endothelial cell delamination;GO:0071642 positive regulation of macrophage inflammatory protein 1 alpha production;GO:0030103 vasopressin secretion |
| TXNRD2 | thioredoxin reductase 2 | GO:0010269 response to selenium ion;GO:0055093 response to hyperoxia;GO:0000305 response to oxygen radical |
| TYMS | thymidylate synthetase | GO:0019860 uracil metabolic process;GO:0006231 dTMP biosynthetic process;GO:0009177 pyrimidine deoxyribonucleoside monophosphate biosynthetic process |
| UGT1A1 | UDP glucuronosyltransferase family 1 member A1 | GO:0070980 biphenyl catabolic process;GO:0006789 bilirubin conjugation;GO:0018879 biphenyl metabolic process |
| URI1 | URI1 prefoldin like chaperone | GO:0010923 negative regulation of phosphatase activity;GO:0035305 negative regulation of dephosphorylation;GO:0010921 regulation of phosphatase activity |
| VKORC1 | vitamin K epoxide reductase complex subunit 1 | GO:0017187 peptidyl-glutamic acid carboxylation;GO:0018214 protein carboxylation;GO:0042373 vitamin K metabolic process |
| ZC2HC1C | zinc finger C2HC-type containing 1C |  |
| ZNF19 | zinc finger protein 19 | GO:0006357 regulation of transcription by RNA polymerase II;GO:0006355 regulation of DNA-templated transcription;GO:2001141 regulation of RNA biosynthetic process |

**Supplementary Table 2** Demographic data of enrolled patients with HCC in this study

| Variable | *n*=62 (western blotting) | *n*=21 (Metabolites analysis) |
| --- | --- | --- |
| Age (years) | 62.7±10.6 | 63.2±10.8 |
| Gender (Male) | 50 (81%) | 17 (81%) |
| Etiology (HBV, HCV, NBNC) | 37, 10, 15 | 13, 4, 4 |
| ICG R15 | 11.6± 8.5 | 11.12± 7.25 |
| AST(IU/L) | 48.6±27.9 | 41.7±20.5 |
| ALT(IU/L) | 41.8±22.2 | 41.6±23.29 |
| Bil (mg/dL) | 0.7±0.39 | 0.67±0.28 |
| ALB | 4.3±0.35 | 4.3±0.34 |
| AFP | 1567.3±8055 | 2984.2±13182.5 |
| AFP>1000ng/ml (number) | 9 (14.5%) | 2 (9.5%) |
| Tumor size (cm) | 6±4.6 | 5.4±3.1 |
| Tumor size >5cm (number) | 26 (41.9%) | 8 (38.1%) |
| Tumor rupture (number) | 5 (8.1%) | 1 (4.7%) |
| Cirrhosis (number) | 23 (37.1%) | 7 (33.3%) |
| Grade I/II: III/IV | 31, 31 | 11, 10 |
| AJCC staging I/II/III and IV | 29, 13, 18, 2 | 14, 3, 4, 0 |
| Major hepatectomy (number) | 24 (38.7%) | 10 (47.6%) |
| Anatomic resection (number) | 24 (38.7%) | 8 (38.1%) |
| Satellite nodule (number) |  |  |
| No | 46 (80.7%) | 16 (76.2%) |
| Single | 3 (5.3%) | 1 (4.7%) |
| multiple | 9 (14.5%) | 5 (23.8%) |
| Vascular invasion (numer) |  |  |
| No | 34 (54%) | 16 (76.2%) |
| microscopic | 18 (28.5%) | 2 (9.5%) |
| Gross | 10(16.1%) | 3 (14.3%) |

**Supplementary Table 3** The information of cell lines used in this study

| **Items** | **Name** | **Source (catalog number)** | **Catalog number** |
| --- | --- | --- | --- |
| cell lines | Hep3B | BioResource Collection and Research Center of Taiwan (BCRC 60434) | BCRC 60434 |
|  | Huh7 | Japanese Collection of Research Bioresources (JCRB 0403) | JCRB 0403 |
|  | PLC5/PRF/5 | Japanese Collection of Research Bioresources (JCRB 0406) | JCRB 0406 |
|  | HepG2 | BioResource Collection and Research Center of Taiwan | BCRC RM60025 |
|  | SNU-475 | American Type Culture Collection (CRL-2236) | CRL-2236 |
|  | SNU-398 | American Type Culture Collection (CRL-2233) | CRL-2233 |
|  | THLE2 | American Type Culture Collection (CRL-2706) | CRL-2706 |
|  | THLE3 | American Type Culture Collection  (CRL-3583) | CRL-3583 |
|  | HEK293 | BioResource Collection and Research Center of Taiwan (BCRC 60019) | BCRC-60019 |

**Supplementary Table 4** Antibodies and reagents used in this study

| **Antibody name** | **Company** | **Cat. No** |
| --- | --- | --- |
| **Western blotting** | | |
| SOX4 | abclonal | a21222 |
| STAT6 | cell signaling | 5397 |
| Actin | Santa Cruz | sc47778 |
| Tubulin | Santa Cruz | sc32293 |
| GAPDH | Santa Cruz | sc47724 |
| Flag tag | cell signaling | 14793 |
| pSTAT6-Y641 | cell signaling | 56554S |
| Calmodulin binding protein (CBP) tag | millipore | 05-932 |
| HA tag | cell signaling | 3724 |
| MTHFD2 | cell signaling | 41377 |
| **Immunoprcipitation beads or Antibody** | | |
| protein G beads | Invitrogen | 10004D |
| M2 beads | Sigma | M8823 |
| streptavidin agarose | Invitrogen | 15942-050 |
| SOX4 antibody | abclonal | a21222 |
| **Immunofluorescence antibody** | | |
| SOX4 | abclonal | A10717 |
| STAT6 | Santa Cruz | sc-271213 |
| Flag tag | Cell Signaling | 14793S |
| Alexa Fluor 488 | Thermo Fisher Scientific | A11001 |
| Alexa Fluor 549 | Thermo Fisher Scientific | A11010 |
| **ChIP seq** | | |
| IgG | Cell signaling | 66362 |
| SOX4 | abnova | PAB14092 |
| STAT6 | cell signaling | 5397 |

**Supplementary Table 5** Primers used in this study

| primer | Sequence (5’- 3’) |
| --- | --- |
| NTAP-SOX4 F | CTGCCCGGGCGGATCCATGGTGCAGCAAACCACAATG |
| NTAP-SOX4 R | CGGTATCGATAAGCTTTCAGTAGGTGAAAACCAGGTTGG |
| NTAP-SOX4 135R | CGGTATCGATAAGCTTCTTCACCTTCTTCCTGGGCCG |
| NTAP-SOX4 227R | CGGTATCGATAAGCTTCCCGCCGCCGCCGCCGCC |
| NTAP-SOX4 397R | CGGTATCGATAAGCTTGGAGGACGAGGAGCCCGAGG |
| NTAP-SOX4 512F | CTGCCCGGGCGGATCCATGGAGAAGGGAGACAAGGTCG |
| Flag-STAT6 F | TAGCCCGGGCGGATCCATGTCTCTGTGGGGTCTGGTCT |
| Flag-STAT6 R | CGGTATCGATAAGCTTCCAACTGGGGTTGGCCCT |
| Flag-STAT6 276F | TAGCCCGGGCGGATCCATGACCCTCGTCACCAGTTGC |
| Flag-STAT6 517F | TAGCCCGGGCGGATCCATGTACTGGTCTGACCGGCTG |
| STAT6 QPCR F | CCTTGGAGAACAGCATTCCTGG |
| STAT6 QPCR R | GCACTTCTCCTCTGTGACAGAC |
| RANKL QPCR F | GCCTTTCAAGGAGCTGTGCAAAA |
| RANKL QPCR R | GAGCAAAAGGCTGAGCTTCAAGC |
| MTHFD2 QPCR F | CTCCTTGTTCAGTTGCCTCTTCC |
| MTHFD2 QPCR R | CTGATCCAAACACATTCGTCCTAC |
| 18S QPCR F | AAACGGCTACCACATCCAAG |
| 18S QPCR R | CCTCCAATGGATCCTCGTTA |
| MTHFD2 ChIP-QPCR F | GAGTTTGACTTCCTCAGCCT |
| MTHFD2 ChIP-QPCR R | CCACTGGCTGTCTGTTGAG |
| STAT6 promoter -1000 | GCTAGCCTCGAGGATATCAGGCCTCTCTGGATCCCTTGG |
| STAT6 promoter R | CCGGATTGCCAAGCTTAGCCCGCTGTTTCCGGCTTC |
| STAT6 promoter -750 | GCTAGCCTCGAGGATATCTTTGTTTCTTGGCGTGTCTCAGTG |
| STAT6 promoter -500 | GCTAGCCTCGAGGATATCGTCAGTGGTCACGAGACCGAC |
| STAT6 promoter -250 | GCTAGCCTCGAGGATATCGGTGACTTTCTGGAGAAAAGCTGA |
| MTHFD2 promoter F | GCTAGCCTCGAGGATATCTTCAACACATTGGCCAGGC |
| MTHFD2 promoter R | CCGGATTGCCAAGCTTGAGCTCTGTGACCCTCTTACCG |
| MTHFD2 promoter -600 | GCTAGCCTCGAGGATATCAATGGTGATTTTCTAACTCCATCA |
| MTHFD2 promoter -300 | GCTAGCCTCGAGGATATCTTACGTTTAATAAAGAAAACCGGTT |
| Nanog Forward | CTCCAACATCCTGAACCTCAGC |
| Nanog Reverse | CGTCACACCATTGCTATTCTTCG |
| SOX2 Forward | GCTACAGCATGATGCAGGACCA |
| SOX2 Reverse | TCTGCGAGCTGGTCATGGAGTT |

**Reference**

1 Pang Z, Lu Y, Zhou G, Hui F, Xu L, Viau C *et al*. MetaboAnalyst 6.0: towards a unified platform for metabolomics data processing, analysis and interpretation. *Nucleic Acids Res* 2024; 52: W398-W406.

2 Pinyol R, Montal R, Bassaganyas L, Sia D, Takayama T, Chau GY *et al*. Molecular predictors of prevention of recurrence in HCC with sorafenib as adjuvant treatment and prognostic factors in the phase 3 STORM trial. *Gut* 2019; 68: 1065-1075.

3 Ianevski A, Giri AK, Aittokallio T. SynergyFinder 2.0: visual analytics of multi-drug combination synergies. *Nucleic Acids Res* 2020; 48: W488-W493.
