# Supplementary material for: SOX4-STAT6-MTHFD2 axis drives hepatocellular carcinoma progression and treatment resistance
Source: Cell Death Dis. 2026 Jan 3;17(1):154. doi: 10.1038/s41419-025-08394-2 (PMC12858837; doi:10.1038/s41419-025-08394-2)
Supplement: Supplementary file 2 — Original data-1 [file 41419_2025_8394_MOESM2_ESM.pptx]

## Slide 1
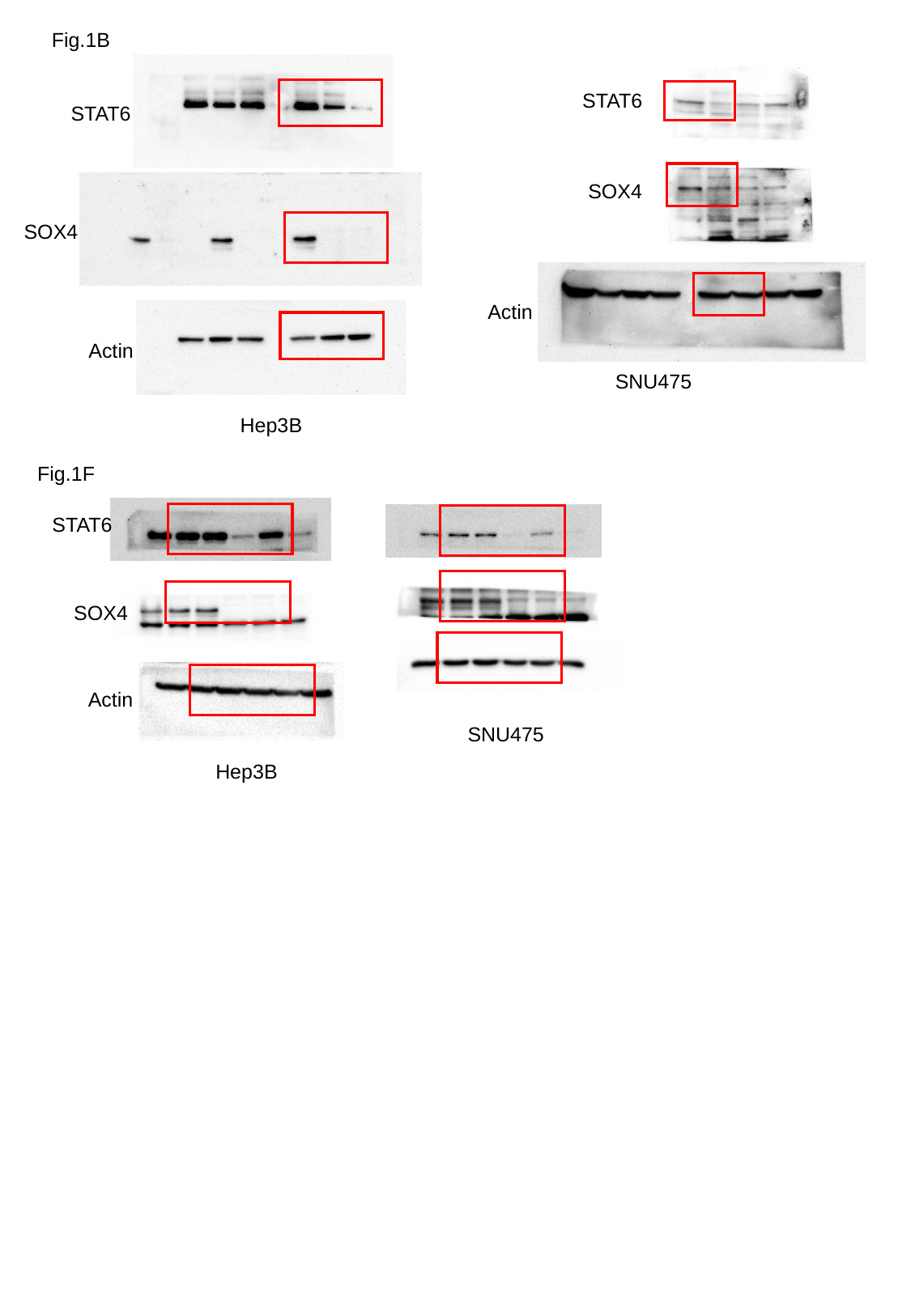

Fig.1B
STAT6
SOX4
Actin
STAT6
SOX4
Actin
SNU475
Hep3B
Fig.1F
STAT6
SOX4
Actin
Hep3B
SNU475

## Slide 2
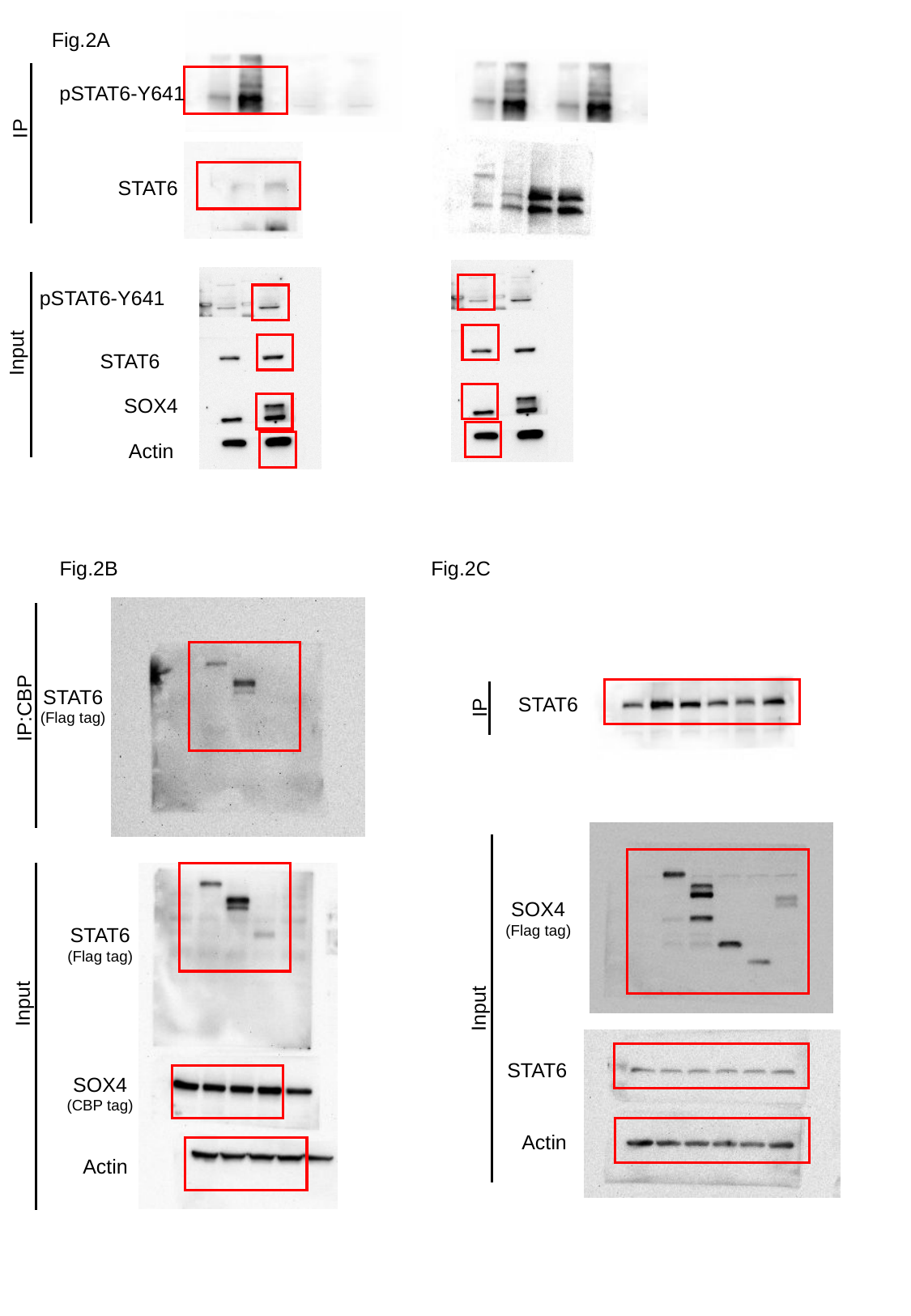

Fig.2A
pSTAT6-Y641
IP
STAT6
pSTAT6-Y641
Input
STAT6
SOX4
Actin
Fig.2B
Fig.2C
STAT6
(Flag tag)
STAT6
IP
IP:CBP
SOX4
(Flag tag)
STAT6
(Flag tag)
Input
Input
STAT6
SOX4
(CBP tag)
Actin
Actin

## Slide 3
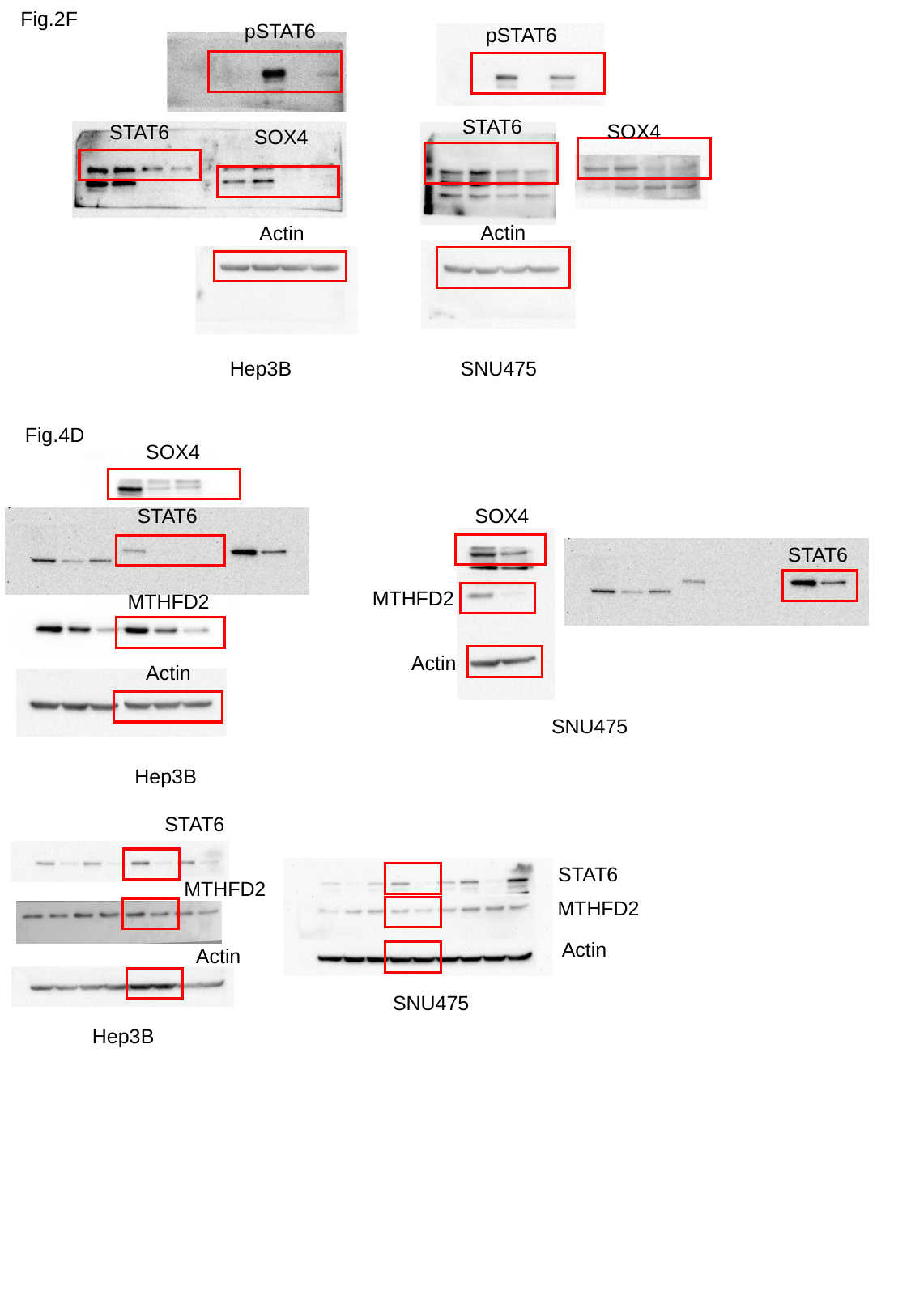

Fig.2F
pSTAT6
STAT6
SOX4
Actin
Hep3B
pSTAT6
STAT6
SOX4
Actin
SNU475
Fig.4D
SOX4
STAT6
SOX4
STAT6
MTHFD2
Actin
MTHFD2
Actin
SNU475
Hep3B
STAT6
STAT6
MTHFD2
Actin
MTHFD2
Actin
SNU475
Hep3B

## Slide 4
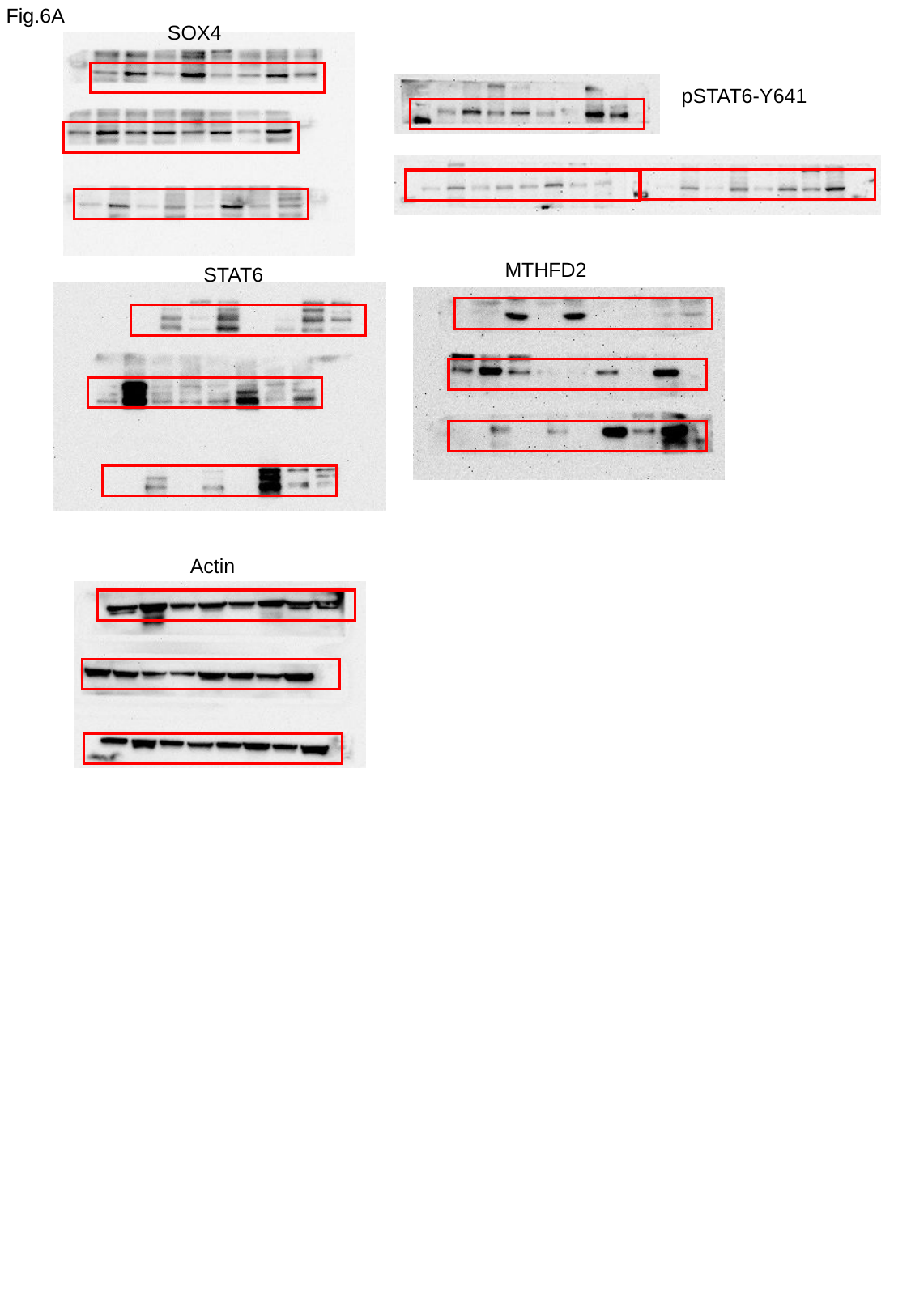

Fig.6A
SOX4
pSTAT6-Y641
MTHFD2
STAT6
Actin

## Slide 5
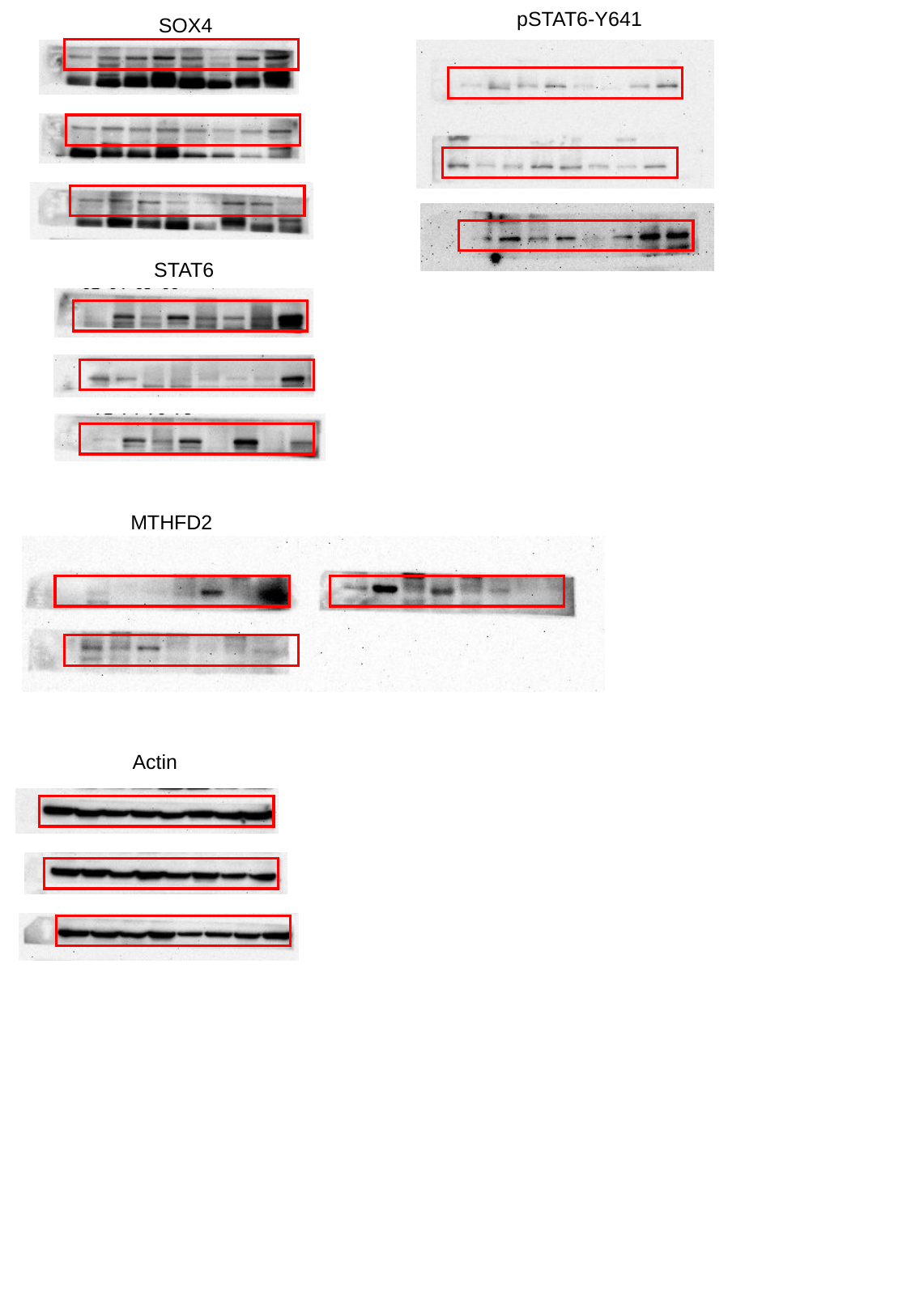

pSTAT6-Y641
SOX4
STAT6
MTHFD2
Actin

## Slide 6
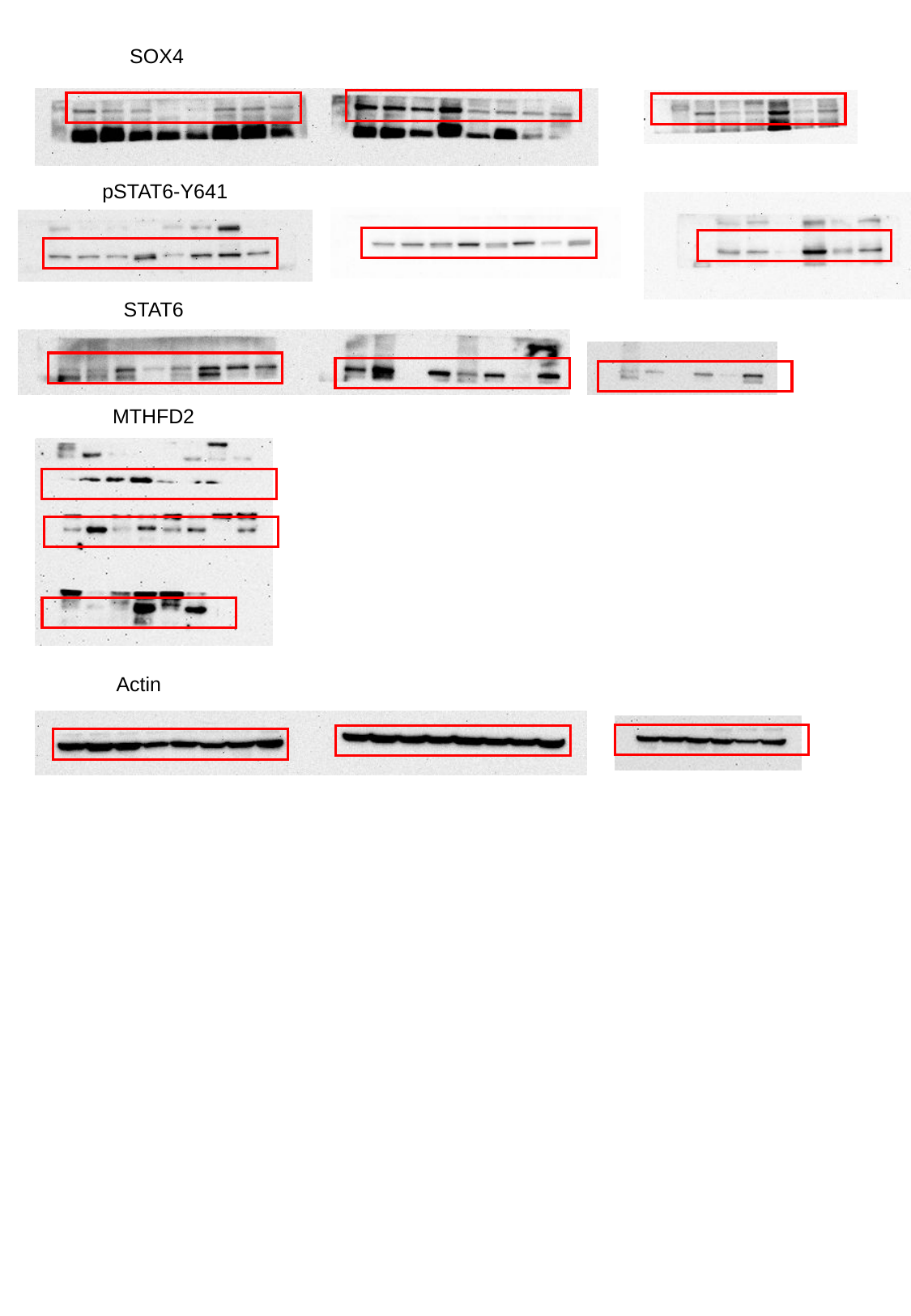

SOX4
pSTAT6-Y641
STAT6
MTHFD2
Actin

## Slide 7
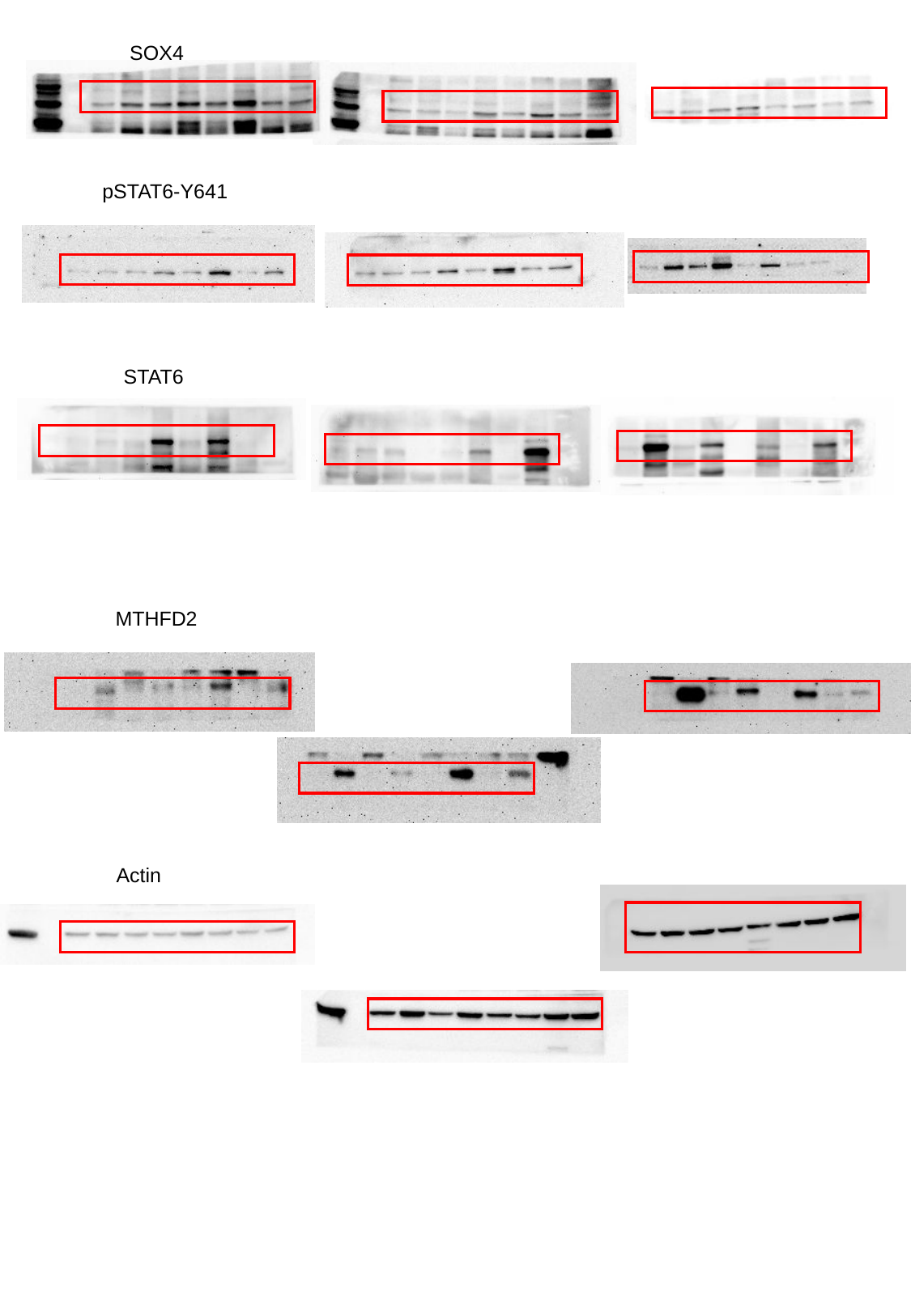

SOX4
pSTAT6-Y641
STAT6
MTHFD2
Actin

## Slide 8
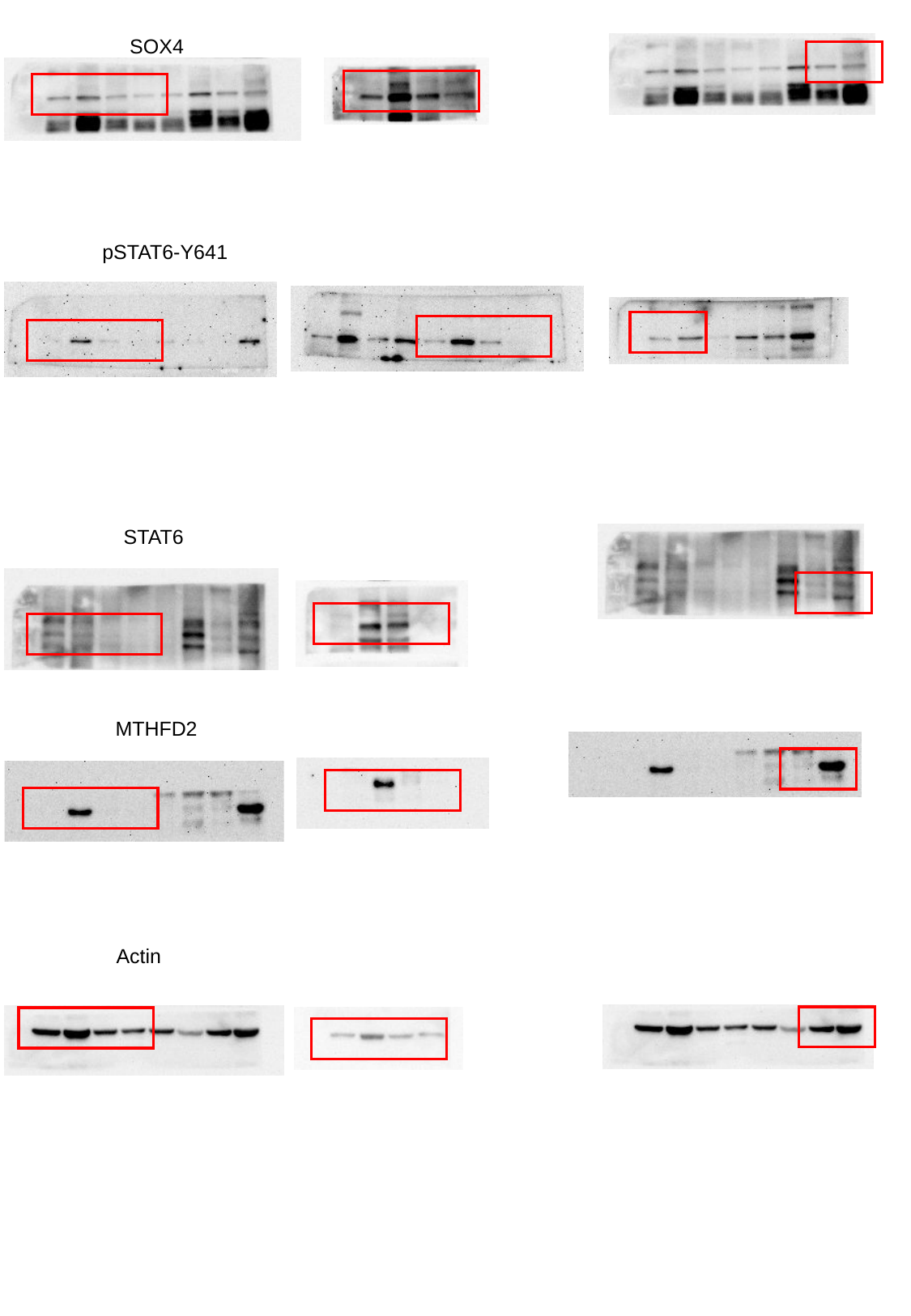

SOX4
pSTAT6-Y641
STAT6
MTHFD2
Actin

## Slide 9
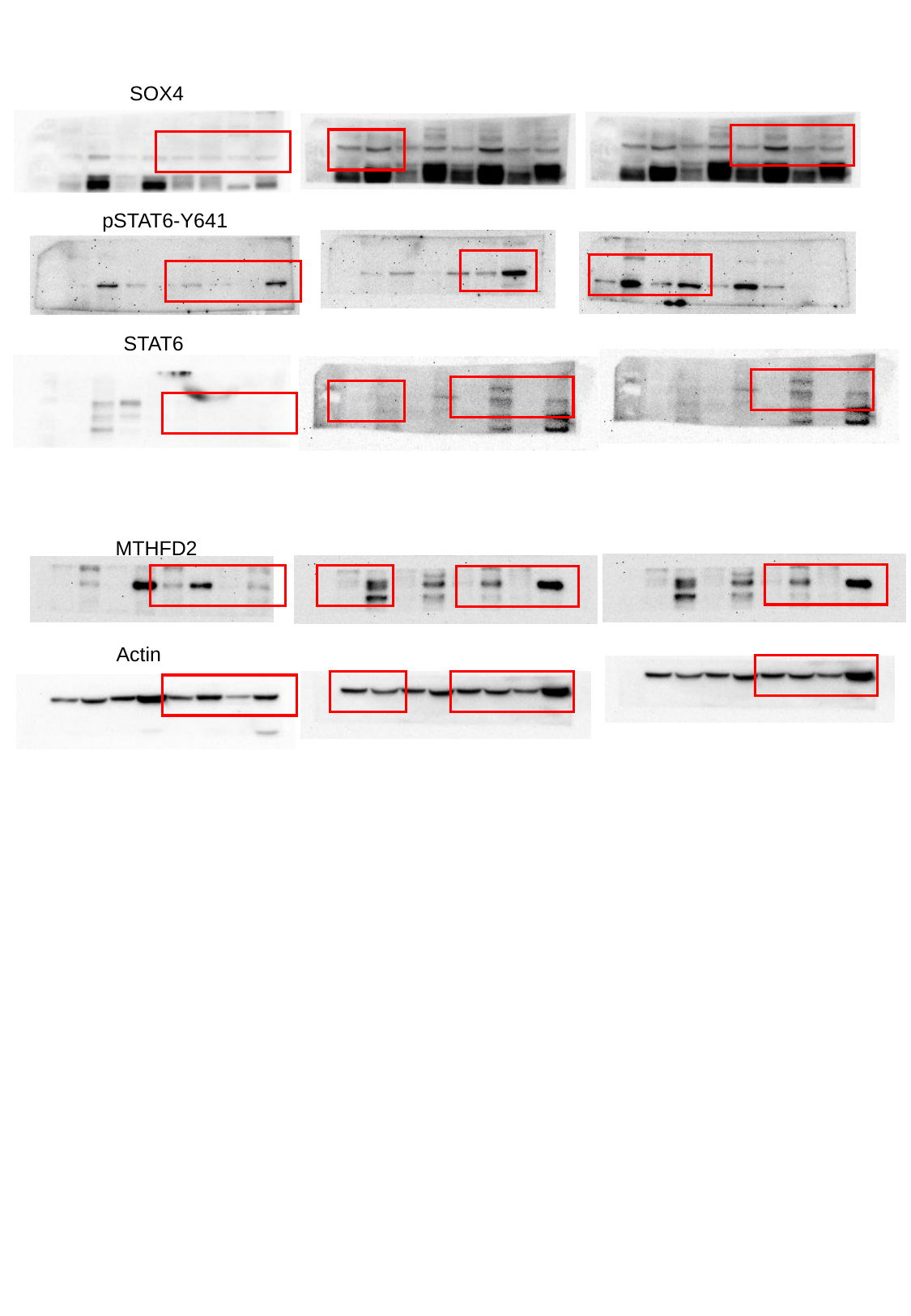

SOX4
pSTAT6-Y641
STAT6
MTHFD2
Actin

## Slide 10
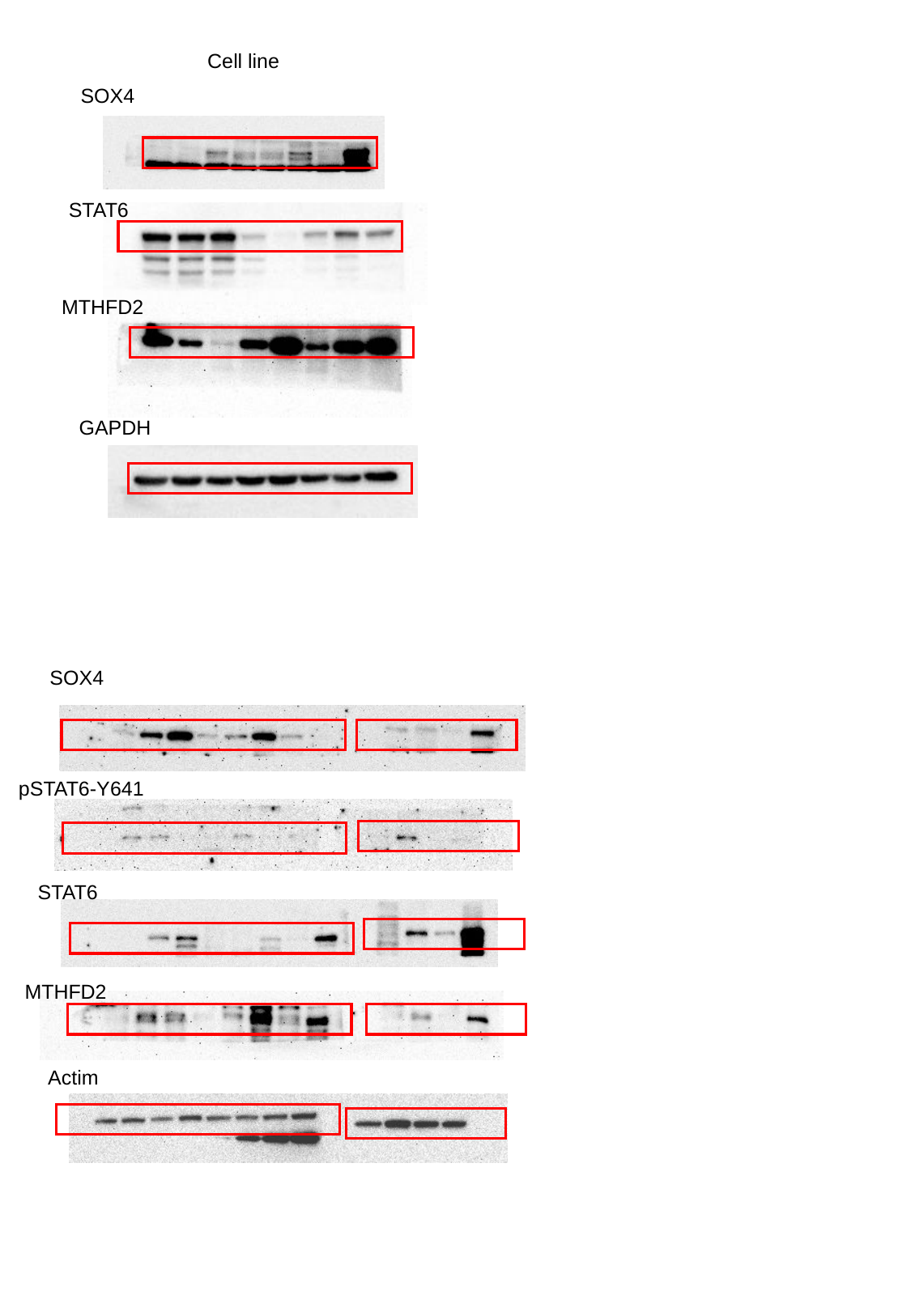

Cell line
SOX4
STAT6
MTHFD2
GAPDH
SOX4
pSTAT6-Y641
STAT6
MTHFD2
Actim
